# Does Vaccine-Induced Maternally-Derived Immunity Protect Swine Offspring against Influenza a Viruses? A Systematic Review and Meta-Analysis of Challenge Trials from 1990 to May 2021

**DOI:** 10.3390/ani13193085

**Published:** 2023-10-03

**Authors:** Sheila Keay, Zvonimir Poljak, Famke Alberts, Annette O’Connor, Robert Friendship, Terri L. O’Sullivan, Jan M. Sargeant

**Affiliations:** 1 Department of Population Medicine, Ontario Veterinary College, University of Guelph, Guelph, ON N1G 2W1, Canada; zpoljak@uoguelph.ca (Z.P.); falberts@uoguelph.ca (F.A.); rfriends@ovc.uoguelph.ca (R.F.); tosulliv@uoguelph.ca (T.L.O.); sargeanj@uoguelph.ca (J.M.S.); 2 Department of Large Animal Clinical Sciences, College of Veterinary Medicine, Michigan State University, East Lansing, MI 48824, USA; oconn445@msu.edu; 3 Centre for Public Health and Zoonoses, Ontario Veterinary College, University of Guelph, Guelph, ON N1G 2W1, Canada

**Keywords:** systematic review, influenza A viruses of swine, influenza vaccines, maternally derived immunity, meta-analysis

## Abstract

**Simple Summary:**

Vaccinated mother pigs can pass vaccine protection (immunity) onto their offspring, but in the case of influenza, the benefit is not clear. Influenza viruses evolve rapidly and can sometimes change their appearance to the point of becoming a new stain that evades a pig’s immune defenses. It is believed that matching the vaccine to the virus is important for protection. To predict if a vaccine is protective it is, therefore, also important to know how closely it matches the influenza strain or strains circulating in the herd. For the first time, we apply the methods of evidence-based medicine to understand if vaccinating sows results in the protection of their piglets from influenza. We were only interested in studies where the researcher knew if the vaccine matched the infecting virus. These types of studies are called challenge trials because researchers controlled both the vaccines used in the mother pigs and the strain of influenza viruses used to infect the piglets. We looked at scientific studies published over twenty years and considered many ways to measure protection. We found piglets from vaccinated sows took a little bit longer to shed the virus if they became infected and that less virus was found in piglets where their mother’s vaccine matched the virus used to infect the piglet. In modern commercial farms, however, piglets are often exposed back-to-back or at the same time to the same, or more often, to more than one strain of influenza. Also, viruses behave differently in herds than they do in small studies because the number of pigs in a herd is many times greater. Because most studies involved simple exposures in small groups of pigs and only one small study looked at a back-to-back exposure with the same strain of influenza, it is difficult to know if our findings can be further extended into the real world. Despite this, the body of research was useful to show the importance of matching. We also learned that additional research is still needed and importantly, that there is room for improvement in how influenza vaccine studies in pigs are reported. Influenza vaccine research is complex in pigs and it is important to understand the type or types of virus strains involved in each study. Future research is needed where researchers are able to identify all infecting strains of influenza and the piglets experience real-world influenza virus exposures.

**Abstract:**

It is unclear if piglets benefit from vaccination of sows against influenza. For the first time, methods of evidence-based medicine were applied to answer the question: “Does vaccine-induced maternally-derived immunity (MDI) protect swine offspring against influenza A viruses?”. Challenge trials were reviewed that were published from 1990 to April 2021 and measured at least one of six outcomes in MDI-positive versus MDI-negative offspring (hemagglutination inhibition (HI) titers, virus titers, time to begin and time to stop shedding, risk of infection, average daily gain (ADG), and coughing) (*n* = 15). Screening and extraction of study characteristics was conducted in duplicate by two reviewers, with data extraction and assessment for risk of bias performed by one. Homology was defined by the antigenic match of vaccine and challenge virus hemagglutinin epitopes. Results: Homologous, but not heterologous MDI, reduced virus titers in piglets. There was no difference, calculated as relative risks (RR), in infection incidence risk over the entire study period; however, infection hazard (instantaneous risk) was decreased in pigs with MDI (log HR = −0.64, 95% CI: −1.13, −0.15). Overall, pigs with MDI took about a ½ day longer to begin shedding virus post-challenge (MD = 0.51, 95% CI: 0.03, 0.99) but the hazard of infected pigs ceasing to shed was not different (log HR = 0.32, 95% CI: −0.29, 0.93). HI titers were synthesized qualitatively and although data on ADG and coughing was extracted, details were insufficient for conducting meta-analyses. Conclusion: Homology of vaccine strains with challenge viruses is an important consideration when assessing vaccine effectiveness. Herd viral dynamics are complex and may include concurrent or sequential exposures in the field. The practical significance of reduced weaned pig virus titers is, therefore, not known and evidence from challenge trials is insufficient to make inferences on the effects of MDI on incidence risk, time to begin or to cease shedding virus, coughing, and ADG. The applicability of evidence from single-strain challenge trials to field practices is limited. Despite the synthesis of six outcomes, challenge trial evidence does not support or refute vaccination of sows against influenza to protect piglets. Additional research is needed; controlled trials with multi-strain concurrent or sequential heterologous challenges have not been conducted, and sequential homologous exposure trials were rare. Consensus is also warranted on (1) the selection of core outcomes, (2) the sizing of trial populations to be reflective of field populations, (3) the reporting of antigenic characterization of vaccines, challenge viruses, and sow exposure history, and (4) on the collection of non-aggregated individual pig data.

## 1. Introduction

### 1.1. Rationale

Research on influenza vaccine-induced maternally-derived immunity (MDI) in piglets has important commercial relevance. Colostrum (predominantly as immunoglobulins) is the piglet’s only source of MDI [1,2] and consumption in the first hours of life is essential for piglet survival [3]. In the United States, the majority of breeding herds are vaccinated against influenza A viruses of swine (IAV-S) [4,5] to protect breeding females (gilts and sows), and through MDI, to confer protection to offspring [6,7,8,9,10]. The benefit of maternal IAV-S vaccination for piglets is, however, questioned [11,12,13,14,15]. Passive immunoglobulin G (IgG) is a known immunosuppressant [1,16] and vaccine failure in both infants and animals has long been attributed to maternally-derived antibodies (MDA) [17,18,19,20]. Protection of young animals in endemic situations is thus complicated by maternal immunity [20]. 

### 1.2. Influenza HA Immunoglobulins (Antibodies)

Two glycoproteins disproportionately shape influenza virus-host interactions and are important determinants of vaccine efficacy; (1) host immunoglobulins (Ig) (mucosal and circulating) and (2) influenza virus hemagglutinin (HA) [21]. HA, located externally on the virus membrane, is configured as a globular head (HA1) and stem (HA2) with multiple structural binding sites (epitopes) for host immunoglobulins [21]; it is the most prevalent and immunologically dominant IAV protein, and is largely responsible for host and tissue specificity [22,23] and for sub-type and strain identity [24,25].

Antibodies produced in response to IAV-S exposure can be neutralizing or non-neutralizing. Neutralizing antibodies prevent the infection, replication, or transmission of the virus, and most, but not all neutralizing antibodies are directed at epitopes within the sialic acid binding site on the head of the HA molecule (HA1) [23,26,27,28,29]. Under specific circumstances, non-neutralizing cross-reactive IgG antibodies have been associated with enhanced disease [26,30,31,32,33,34]. In pigs and ferrets, enhanced disease known as vaccine-associated enhanced respiratory disease (VAERD) has been consistently reproduced as a severe lung pathology in IAV challenge trials involving the use of sub-type heterologous killed vaccines [34,35,36].

Influenza vaccine protection is largely dependent on the antigenic match between the HA component(s) of the vaccine with the HA of the challenging virus(s) [22,23,27]. In modern commercial production systems, genetically and antigenically diverse viral sub-types and strains co-circulate within production stages [37,38,39], across stages [14,40,41,42,43], and across regions [44,45]. Frequent influenza virus mutations with subsequent changes in epitope antigenicity, and introduction of new sub-types or strains, means vaccine-induced protection is short-lived unless vaccines are updated [28,29].

### 1.3. Measures of Influenza Vaccine Protection

Vaccine protection can be assessed directly using specific endpoints such as the detection of infection, or non-specific endpoints such as clinical disease. Objective and subjective measures of clinical disease are important for post-marketing evaluation of vaccine effectiveness [46,47]. Hemagglutination inhibition (HI) assay titers are indirect measures of protection and the gold standard immune correlate of protection (CoP) against influenza [48,49]. Standardized monospecific antiserum reagents are used in the HI assay to sub-type influenza A viruses [48]. Depending on the HI assay configuration, it can be used to sub-type viruses or to determine titers of anti-HA antibodies in serum samples (i.e., HI titers) [50], and assay specificity is limited by assay reagent homology with the respective virus or antibodies in samples [50,51]. Homology is a phenotypic characterization of the match of virus HA antigens (and epitopes) with host immunoglobulins [51].

### 1.4. Influenza Virus Subtypes, Strains, and Homology

Of the 18 sub-types of influenza viruses identified (H1-H18), H1 and H3 circulate in swine populations globally as H1N1, H1N2 and H3N2 [47,50,52,53,54,55,56], with sub-types H1 and H3 further characterized by strain [45,51]. In standard HI assays, and depending on assay configuration, homology is determined by the degree of cross-reactivity of viral antigens with reference immunoglobulins (antiserum), or of host immunoglobulins with reference viral antigens [51]. The HI assay is biased towards antigenically dominant HA epitopes [51,57] and detection of a subtype or strain to which the pig has not been exposed does not generally occur unless samples contain antibodies directed against highly conserved antigens (i.e., conserved across different viruses) [29,51]. It is also possible to observe little to no cross-reaction between viruses that are of the same sub-type but are otherwise antigenically different; such viruses are described as sub-type homologous/strain heterologous [45,51]. Characterization of sow exposure history, vaccine, and challenge virus homology by HA subtype and by strain was, therefore, emphasized throughout this review.

Maternally-derived immunity substantively impacts piglet survival but the impact of IAV-S vaccine-induced maternally-derived immunity is not clear. Epitope affinity and immunodominance of immunoglobulins in colostrum is a function of the sequential timing and totality of each sow’s prior IAV-S exposure(s) [58,59]. In the virally dynamic setting of the field, defining the nature of virus exposure is challenging; therefore, we systematically reviewed challenge trial research such that piglet virus subtype and strain exposure could be defined. Where possible, up to six direct and indirect outcome measures were synthesized for inclusion in meta-analyses to determine IAV-S protective impacts of sow vaccination on piglets. This is the first time methods of evidence-based medicine have been applied to influenza vaccine research in swine to conduct a systematic review and meta-analysis.

## 2. Methods:

### 2.1. Protocol and Registration

The protocol was posted on 31 March 2021, in advance of study commencement, on the University of Guelph Atrium URI: https://hdl.handle.net/10214/24720 (accessed on 20 September 2023), and on the website Systematic Reviews for Animals & Food (SYREAF), http://www.syreaf.org (accessed on 20 September 2023).

### 2.2. Objectives

The objective of this review was to answer the question:

“Does vaccine-induced maternally-derived immunity (MDI) protect swine offspring against challenge with influenza A virus?”.

### 2.3. Eligibility Criteria

Eligible publications were published since 1990, from any geographic location, and available as an English language full text. Eligible question elements included:

Population = Swine dams (intervention population) and their offspring (population measured for outcomes).

Intervention (treatment) = IAV-S pre-farrowing vaccination of dams using any vaccine platform or schedule. Vaccinated dams additionally exposed naturally to IAV-S were eligible. Both IAV-S vaccinated and non-vaccinated offspring (i.e., piglets were also vaccinated) of IAV-S vaccinated sows were eligible. Assuming passive transfer of colostrum to offspring, offspring from vaccinated dams were hereafter referred to as MDI positive.

Comparisons = All comparisons were control arms comprised of dams with no prior IAV-S exposure as stated by the authors. Control arms (i.e., IAV-S negative and non-vaccinated dams) where the piglet offspring were subsequently vaccinated against IAV-S were eligible for data collection for study characterization but were omitted from all analyses. Studies were excluded if control group animals (i.e., IAV-S negative dams and their non-vaccinated offspring) were IAV-S positive (serologically or by virus detection) prior to the piglet challenge. Based on an assumption of no passive transfer of immune factors against IAV-S from control dams to their offspring, hereafter control group offspring from IAV-S negative dams were referred to as MDI negative.

Outcomes (6): At least 1 of the following 6 outcomes were measured in the offspring:

Direct measures of protection–specific endpoints (from nasal swab samples):

1. Virus titers
2. Virus detection (incidence risk, time to shedding)
3. Duration of virus shedding (time to stop shedding)

Indirect measure of protection–correlate of protection (CoP) (from serum samples):

4 Hemagglutination inhibition (HI) titers

Direct measures of infection–non-specific endpoints (clinical observations):

5 Average daily gain (ADG)
6 Coughing (incidence risk).

Study designs: Only controlled trials involving purposeful IAV-S challenge of the offspring of IAV-S vaccinated dams (treatment population) and of IAV-S negative dams (control population) were eligible.

### 2.4. Information Sources and Search Strategy

Previously, 376 potentially relevant publications were identified from a scoping review of primary IAV-S vaccine research in pigs (from 1990 to 2018) [60]. To update the list of relevant publications, sources were searched as follows:

Platforms (databases) searched 13 April 2021:

CAB Direct (CAB Abstracts and Global Health-2018-current), PubMed (MEDLINE)

Web of Science (The Science Publication Index, Clarivate Analytics, 2018-current-multiple databases), 

ProQuest (Agricola–USDA National Agricultural Library 2018-Current),

ProQuest (Dissertations & Theses A&I: Health & Medicine Full Text (2018–2021)).

The search strategy for the previously identified 376 publications is detailed in Keay et al. (2020) [60]. The same search string was applied but modified to include terms for immunization for the updated search (detailed in Text S1). For Web of Science the search string was: ((TS = (pork OR swine OR “Sus scrofa” OR pig OR pigs OR piglet OR piglets OR gilt OR gilts OR boar OR boars OR sow OR sows OR hog OR hogs OR “weaner pig” OR “weaned pig$” OR “feeder pig$” OR feeder OR feeders OR “finisher pig$” OR “finisher hog$” OR porcine OR “market-weight” NOT “guinea pig$”) AND TS = (influenza OR IAV OR IAV$ OR flu OR SIV OR “H3N2” OR “H1N1” OR “H1N2” OR “H3N1” OR “H2N3”) AND TS = (immunize OR immuniz$ OR immunise OR immunis$ OR immunoprophylaxis OR intervention$ OR vaccinate OR vaccinat$ OR vaccine$ OR vaccine))) AND LANGUAGE: (English) Indexes = SCI-EXPANDED, SSCI, A&HCI, CPCI-S, CPCI-SSH, ESCI Timespan = 2018–2021.

Grey literature sources searched 7 April 2021:

The American Association of Swine Veterinarians (AASV) Online Information Library http://www.aasv.org/library/swineinfo/ (accessed on 20 September 2023), includes conference proceedings from 2018 to current for the AASV Annual Meeting, the AASV Pre-Conference Seminars, the International Pig Veterinary Society Congress (IPVS), the Allen D. Leman Swine Conference, and the ISU Swine Disease Conference for Swine Practitioners, and was searched using search term ‘influenza’.

### 2.5. Study Selection

Publications from the search were screened for eligibility at two levels by two reviewers working independently using two forms (Level 1 and 2) constructed in Distiller SR (© 2023 Systematic Review Software by Evidence Partners). Forms were pre-tested (Level 1 on a sample of 100 citations, Level 2 on 10 journal articles) by two reviewers and amended by consensus. Relevance screening questions for Level 1 (title/abstract), and Level 2 (full text) are detailed in Text S2. Conference proceedings were excluded if less than 500 words or if data were also published as a dissertation or journal article. Likewise, dissertations or theses were excluded if data were published in a journal article.

### 2.6. Data Collection Process

Citations were deduplicated using EndNote reference management software (© 2023 Clarivate Analytics) and Distiller-SR software (© 2023). Relevance screening and study characterization were conducted in duplicate by two reviewers working independently in Distiller-SR. Data for quantitative synthesis were extracted by a single reviewer using Microsoft Excel (2013). The author from an eligible publication was contacted to confirm reported data were the same as that referenced in two other publications [61,62,63]. No other authors were contacted. Data presented in graphical format were extracted using the free online software WebPlotDigitizer, Version 4.5 [64].

### 2.7. Data Items

Trial characteristics extracted included sow IAV-S exposure history, IAV-S vaccine program details for sow and if applicable for piglets (vaccine platforms, antigenic composition, dose, timing, and method of delivery), piglet ages at weaning, timing and method of challenge, challenge virus type, subtype, and or strain, and piglet age at each sampling period. All vaccines were characterized as homologous, heterologous, or as not defined in reference to the challenge virus. Assigned homology was as stated by authors, inferred from provided antigenic details, or was identified as not defined if details were not reported. Multivalent commercial vaccines were characterized as sub-type homologous if at least one of the composite strains matched the challenge virus sub-type. Reported occurrences of VAERD were also noted.

### 2.8. Risk of Bias within Studies

The risk of bias was assessed for each study at the outcome level by a single reviewer using a previously constructed form in DistillerSR that was based on the Risk of Bias tool ROB-2.0 [65] but modified for the evaluation of swine trials [66]. The risk of bias was assessed in five domains: bias from the randomization process, deviations from intended treatments, missing outcome data, measurement of the outcome, and selection of reported results.

### 2.9. Outcome Effect Sizes

Most outcomes were reported as arm-level effects and the calculation of effect sizes was required for inclusion in meta-analyses. Control arms with MDI-negative but IAV-S vaccinated offspring were excluded. To avoid unit of analysis issues (i.e., double counting) the remaining controls were divided by the number of treatment arms with each fraction and then used to calculate effect sizes for each arm. Effect sizes were calculated as described in the following four sections (Section 2.10 to Section 2.13).

### 2.10. Virus Titers–Standardized Mean Differences (SMD)

Virus titer data (log titers) were reported in different units of measure depending on the method of virus quantification (e.g., PCR (rRT-PCR) or cell culture isolation); therefore, effect sizes were calculated from mean log virus titers as standardized mean differences (SMD) using the formula for Hedge’s g that includes a bias correction for small samples sizes [67]. The frequency and timing of nasal swab collection post-challenge differed from study to study, as did the number of treatment arms included in each comparison. A composite effect size was, therefore, calculated for use in meta-analyses. Formulas for combining mean differences across treatment groups and then across multiple time-points were applied as per Borenstein [67] and are detailed in Text S3. Effect size calculations were conducted in Excel. Briefly, SMDs were collapsed first across all treatment arms including concurrent homologous vaccination of piglets (regardless of vaccine platform or dosing schedule), and then across repeated time-points where an adjustment was made to account for the correlation of outcomes from time point to time point; as a sensitivity analysis, the correlation adjustment between repeated measures over time was conducted using an assumed high correlation (0.75) and a low correlation (0.2). Meta-analysis was conducted using each adjustment. Meta-analysis of SMD virus titer data is detailed in Step 01 of Text S4.

### 2.11. Duration of Virus Shedding–Mean Differences (MD), Hazard Ratios (HR)

The duration of virus shedding was measured as two distinct periods; (i) time-to-shedding measured as the mean number of days from the time of challenge until the virus was detected, and (ii) time-to-stop shedding, calculated for infected piglets only, as the mean number of days that infected pigs shed virus during the sampling period. Effect sizes were calculated as mean differences (MD) and hazard ratios (HR) for each period. The hazard was the instantaneous risk during the study period of challenged piglets becoming infected and beginning to shed the virus. In studies where sufficient pig-level data were available, the number of days until animals experienced the outcome of interest was extracted into Excel. The outcome of interest was days to first testing positive post-challenge, or, once infected, days until they no longer tested positive or was censored from the study (i.e., when they died or the study ended before a change in shedding status was detected). Data were analyzed using Cox’s proportional hazard model available in the R survival package (Step 03 in Text S4) with resulting hazard ratios used as inputs for meta-analyses (see Step 4 in Text S4). In addition, individual-level survival data were used to determine the mean period of interest and standard errors for each treatment arm and these were used to determine the MD of periods as inputs for meta-analysis (see Step 05 in Text S4).

### 2.12. Incidence Risk of Infection–Relative Risk (RR)

Meta-analysis was conducted on the relative risk (RR) of infection (see Step 06 in Text S4) using reported effect sizes and measures of dispersion, or if not reported, as calculated in R provided sufficient details were reported to calculate group proportions of infected offspring post-challenge.

### 2.13. Weight Gain, HI Titers, and Coughing–Mean Differences (MD), Relative Risk (RR)

The effect size was the mean difference (MD) for outcomes of ADG (kg) and of baseline mean HI titers at challenge (see Steps 07 and 08, respectively, in Text S4). Group level mean HI titers for each study arm were extracted at each time point sampled using WebPlotDigitizer [64], log-transformed for the calculation of geometric mean titers (GMT) [68], and converted to HI scores (i.e., dilution numbers) using the formula as per Kitikoon et al. 2006 [69]: reciprocal GMT = 2*^n^* × 5 where *n* = the dilution number. Baseline mean HI titers were measures of piglet passive humoral protection at the time of challenge and, where applicable, from active responses to piglet vaccination. All control groups had mean GMT titers of less than 1. For qualitative comparison of each study arm, mean HI scores were jointly plotted by piglet age at sampling (weeks) as stacked graphs using the Excel graphics add-in package Peltier Tech Charts for Excel (Version 4.0) [70]. Coughing outcome was the incidence risk of coughing in each group post-challenge. Reported proportions of pigs coughing per treatment group post-challenge were extracted in Excel and RR was calculated as the effect size for meta-analysis.

### 2.14. Synthesis Methods

All meta-analyses, tests for heterogeneity, assessment for publication bias, and generation of effect sizes when required, were conducted in 8 steps in R (Version 4.1.1) [71] using RStudio [72] applying R packages metaphor (Version3.0–2) (rma and escalc, metabin functions), and survival (Version 3.2–13). R program code is detailed in Text S4 and corresponding raw data are available in Table S1. The use of random effects meta-analysis was decided *a priori* as the true effect was expected to differ between studies due to study heterogeneity. I^2^ was used to describe heterogeneity. Heterogeneity was considered small, moderate, or high for I^2^ values of 25%, 50%, and 75%, respectively, [73] and interpreted in consideration of its 95% uncertainty interval [74,75].

### 2.15. Additional Analysis

Vaccine platform and antigenic composition were assumed sources of between-study heterogeneity; therefore, a priori sub-group analyses of each of four factors were considered to explore heterogeneity; (i) maternal vaccine platforms (i.e., whole inactivated killed vaccine (WIV), live attenuated influenza A vaccines, or other novel platform types), (ii) presumed homology of conferred MDI with the challenge virus(s), (iii) inclusion of concurrent piglet vaccination, and (iv) homology of concurrent piglet vaccines with challenge virus(s). Data supported sub-group analysis by MDA homology only; maternal vaccines were exclusively WIVs, and there were too few applicable studies to complete sub-group meta-analysis by piglet vaccine platforms, or by piglet vaccine homology. Posteriori, sensitivity analysis was conducted by removing single effect sizes judged in forest plots as outliers from the meta-analysis to assess outlier impact on summary effect measures.

### 2.16. Meta-Bias

Where meta-analyses included 10 or more treatment-control comparisons, publication bias was assessed using funnel plots generated in R (using R function ‘funnel’) and missing studies estimated by Duval and Tweedie’s trim and fill method [76] to correct for this bias (using R function ‘trimill’).

### 2.17. Confidence in Cumulative Evidence

Grading of Recommendations Assessment, Development, and Evaluation (GRADE) is an approach to rate the quality of evidence in systematic reviews and to grade the strength of recommendations. GRADE involves classifying evidence into one of four levels of confidence (high, moderate, low, and very low) [77] based on the assessment of the risk of bias, inconsistency of results, indirectness of evidence, imprecision, and reporting (publication) bias. Confidence was downgraded by a level for each serious limitation for any factor, and by two levels if there was a very serious limitation. The final output of the GRADE process was an evidence profile and from which, a Summary of Findings table [77,78]. A single reviewer applied the GRADE approach producing and evidence profile that was reviewed and discussed with two other reviewers to reach, through consensus, a final decision on ratings.

### 2.18. Protocol Deviations

1. Original eligible comparator groups included offspring from dams vaccinated against IAV-S using a vaccine that differed from the treatment vaccine. Prior to completion of level 2 relevance screening it became apparent that too few relevant studies would be available for multiple treatment comparisons, and therefore, the comparison group (i.e., control group) eligibility criteria was changed to be offspring from IAV-S negative dams only.
2. A single reviewer extracted data for meta-analysis and for assessment of risk of bias.
3. The risk of bias form was not pre-tested in consideration of a prior application to swine research syntheses [66].
4. Posteriori, a sensitivity analysis was conducted to remove a single effect size when an outlier effect size was observed in the meta-analysis.
5. All a priori outcomes were considered primary outcomes (i.e., none was considered a secondary outcome).
6. Posteriori, data items for study characterization were expanded to identify studies investigating VAERD as a stated objective.

## 3. Results

### 3.1. Study Selection

**Figure 1:**
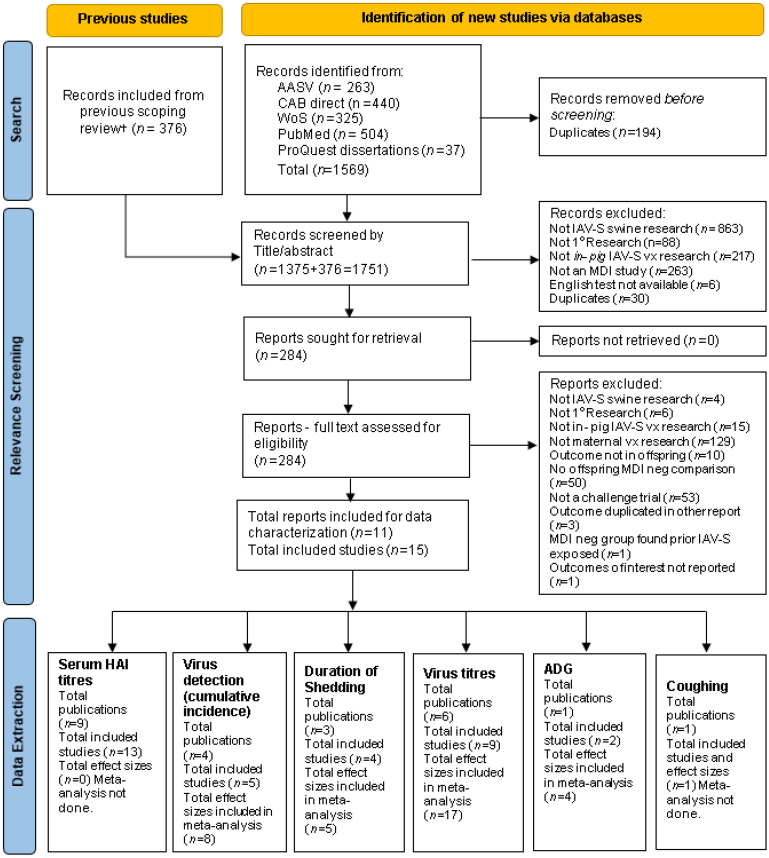
PRISMA flowchart: Flow of citations through search and relevance screening processes to identify IAV-S vaccine-induced MDI challenge trial research involving swine offspring from literature published between 1990 and 2021; PRISMA = Preferred Reporting Items for Systematic Reviews and Meta-Analyses; WoS = Web of Science; AASV = AASV Swine Information Library; MDI= IAV-S vaccine-induced maternally-derived immunity; in-pig = pig level study unit; ^†^ Publications identified in prior scoping review [60]; Effect size defined as difference in outcomes in MDI-positive piglet offspring (treatment group) versus MDI-negative piglet offspring (control group); Each publication may include >1 study and each study may include >1 treatment-control comparison (i.e., multi-armed studies); PRISMA flowchart template modified from Page et al. [84].

Citation inclusion is detailed in the PRISMA flowchart in Figure 1. Of the 376 citations brought forward from the scoping review, 11 publications (15 studies) were included for data extraction. Notable exclusions were trials with no IAV-S negative dams as controls [79,80,81], and field studies [10,12,80,81,82]. A single publication (1 study) (Pyo et al., 2015) [83] was identified on relevance screening as the only study that met all eligibility criteria with the exception that none of the six outcomes of interest were measured in offspring (see Text S2, Level 2 relevance screening question 8). No outcome data were extracted but for completeness, it was included in descriptive charting of study characteristics (i.e., 12 publications, 16 studies). Of the additional 1375 citations identified in the updated search, none were eligible.

### 3.2. Study Characteristics

#### 3.2.1. Selected Study Parameters

**Figure 2:**
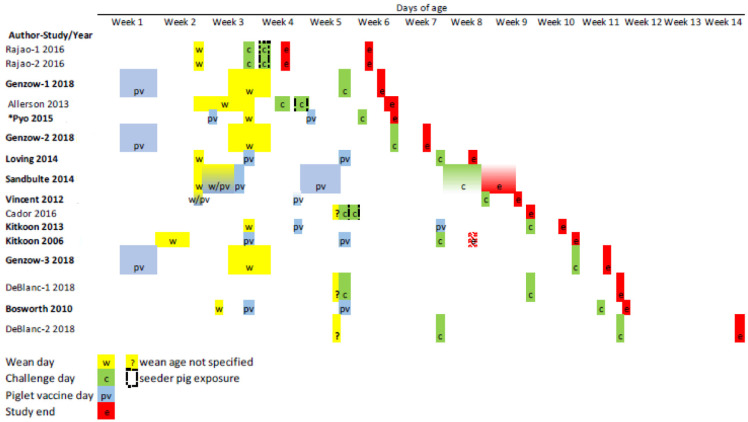
Piglet offspring age (weeks) at occurrence of important study events for each of 16 IAV-S vaccine-induced MDI challenge trials [61,69,83,85,86,87,88,89,90,91,93,94]. *Pyo et al. [83] included descritptively only but no outcome data were extracted.

Study parameters are summarized in Figure 2 and Table 1. The research was funded by governmental organizations [11,61,83,85,86,87,88,89], pharmaceutical companies [69,90], or the source was not reported [91,92]. Data was extracted for meta-analyses from 11 eligible publications inlcusiive of 15 challenge trials, where more than 1 trial was reported in some studies [88,89,93] involving 26 treatment arm comparisions versus a control where several trials included multiple treatment arms [61,85,86,89,90,94] (see Table 2). Nasal swabs were collected in 14 of 15 studies for the detection, isolation and/or quantification of virus, and serum samples for HI assays were collected in 11 studies. Sampling occurred multiple times in the majority of studies with type and collection periodicity varying considerably across studies (see Table S2). Population characteristics and the timing of study events (ages at weaning, piglet vaccination, challenge, and at study end) are summarized in Table 1, and study events are shown sorted by the length of study periods in Figure 2. Age at first challenge ranged from 21 days of age (doa) [88] to greater than 10 weeks [85,89] (Table 1, Figure 2), and post-challenge observation periods ranged from 4 to 48 days [85,89] with almost a third of all studies concluding 5 or fewer days post-challenge (Table 1, Figure 2).

Studies are ordered by length of the study period; events include wean age (yellow w), piglet age at vaccination (blue pv), age at IAV-S challenge (green c) and study end (red e); Author-Study/Year = Primary author-trialnumber (for studies reporting >1 trial)/year of publication; author names in bold = studies with concurrent IAV-S vaccination of offspring; squares with incomplete shading equal studies where event occurrence extended over more than one week; green cells outlined with black dashed line identifies the challenge of offspring via seeder pigs (i.e., indirect challenge). Kitikoon 2006 week 8 cell shaded with red hatched e = study ended for a portion of offspring.

#### 3.2.2. Maternal IAV-S Vaccination Schedules

**Figure 3:**
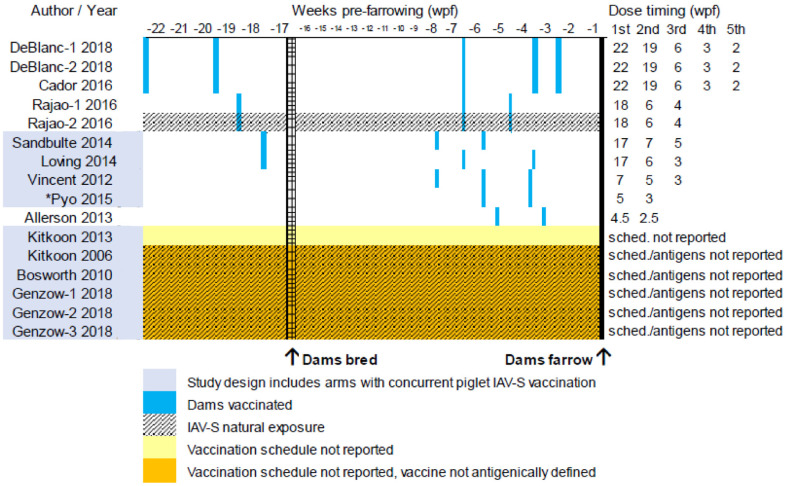
Maternal IAV-S exposure history for sows in 16 vaccine-induced MDI challenge trials: Pre-farrowing IAV-S vaccination schedules and natural virus exposure [61,69,83,85,86,87,88,89,90,91,93,94].

Maternal vaccination schedules are summarized in Figure 3. Maternal and piglet vaccine composition, dosing, and antigenic match in comparison to challenge viruses are detailed in Table S3 and Table S4, respectively. Reporting of vaccination schedules in publications ranged from sparse and non-specific (i.e., an undefined commercial vaccine administered within the 18 months prior to farrowing [93]) to highly detailed (i.e., administration of 5 doses of antigenically defined vaccines at specific weeks within the 22 weeks prior to farrowing [89,91]). Vaccination schedules were not reported in 6 of 16 studies and maternal vaccine composition was not antigenically defined in three publications (5 studies) [69,85,93]. Dams were naturally exposed in six studies [69,85,88,93] and of which natural virus exposure was characterized in one of the six [88].

Studies ordered by earliest pre-farrowing timing (weeks) for vaccination of dams (control group dams were not vaccinated); MDI = vaccine-induced maternally derived immunity; vertical blue lines indicate week pre-farrowing of maternal IAV-S vaccination event, absent blue lines means sow vaccination schedule was not reported; numbers in the columns on the right-hand side equal the week prefarrowing when each vaccination event occurred; Dam = maternal sows/gilts; farrow = piglets born; black arrows indicate the timing of dams bred and of offspring born where week 0 = farrowing date; shading with black horizontal lines vaccinated = dams exposed naturally to IAV-S; *Pyo et al. [83] included descritptively only but no outcome data were extracted.

#### 3.2.3. Vaccine Homology (Sub-Type/Strain) as Referenced to Challenge Viruses

Vaccination schedules and antigenic composition of IAV-S vaccine strains in reference to respective challenge viruses are detailed in Table S3 (maternal vaccines and schedules) and Table S4 (concurrent offspring vaccines and schedules). Where defined, maternal vaccines were sub-type homologous but varied by strain homology. All sub-type and strain antigenic combinations employed across study arms are tabulated in Table 2. Eleven (11) of 20 potential combinations of antigenic matches were identified across 84 study arms involving 1002 offspring, approximately a quarter of which (22/84 arms, 266 offspring) were MDI-negative control arms. Seventeen (17) control study arms with 142 MDI-negative but IAV-S vaccinated offspring were excluded from analyses. Investigation of MDI that was subtype heterologous was not conducted. Antigenic mismatch, or heterology, was investigated at the level of the virus strain only (i.e., strain heterologous) in studies that involved the investigation of the immunologic phenomenon of VAERD. 

For treatment arms, 8 of 16 potential combinations of antigenic matches (of MDI to the challenge virus) were applied across 45 study arms and involved 594 offspring; approximately 60% (369/594) were non-vaccinated offspring and were distributed across three possible treatment arm combinations (Table 2 combination No. 1,2,3). Of the 225 offspring that were vaccinated, they were distributed across treatment arms with five different combinations of antigenic matches (Table 2, combination No. 6,7,9,12 and 13).

### 3.3. Risk of Bias within Studies

Risk of bias (RoB) assessments by outcome are summarized in Table S5. There were some concerns across all outcomes as few details were provided in most studies on any randomization processes employed (domain 1). There was a low likelihood of bias due to assessor awareness of dam vaccination status where outcomes were objectively measured. Publication text supporting the risk of bias assessments by study, by ROB domain, and by outcome is detailed in Table S6.

### 3.4. Direct Measures of Infection–Specific Endpoints

Methods for detecting and quantifying virus from nasal swab samples, collected in all studies but one [90], varied across studies by timing and frequency and by the assay employed (see Table S2).

### 3.5. Outcome 1: Virus Detection (Incidence Risk)–Risk Ratio (RR)

**Figure 4:**
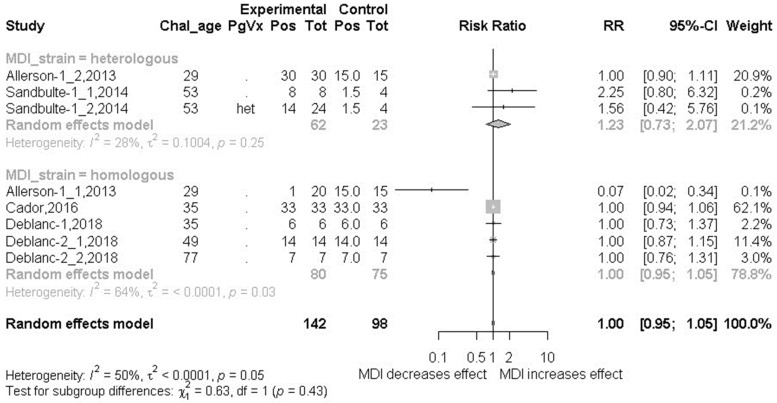
Sub-group meta-analysis forest plot of effects of IAV-S vaccine-induced MDI on relative risk (RR) of infection in IAV-S challenged piglets: sub-grouping by sow vaccine antigenic homology with challenge virus. Study = MDI comparisons at the level of the trial and at the level of the treatment arm comparisons versus a control (in cases where trials include multi-arm comparisons); studies are label following the convention of [Author]-[Trial #]_[treatment arm comparison #], [year of publication]; for study citations refer to Table 1 as listed by author and year of publication. Meta-analysis sub-grouped by MDI strain where MDI_strain = antigenic match of the maternal vaccine composition to the challenge virus (heterologous or homologous); MDI = maternally-derived immunity; MDI-positive = experimental (treatment) groups of offspring of IAV-S vaccinated dams; MDI-negative = control groups of offspring from non-vaccinated, IAV-S negative control dams; studies with concurrent IAV-S vaccination of piglets (Piglet Vx) were identified similarly as strain homologous (hom) or heterologous (het) with the challenge virus; effect = incidence of infection; effect size is the risk ratio; solid vertical line at 0 equals the point of no difference in effects of MDI-positive versus MDI-negative groups (i.e., log risk ratio = 0) and points to the right of the line indicate MDI increases the effect in the treatment group; I^2^ 95% uncertainty interval (lower bound, upper bound) = (0.00, 77.5); effect sizes are represented by squares with size proportional to their weighted contribution to the summary effect measure; weighted contribution is shown as a % in columns on the right; summary effects sizes are represented by diamonds [61,89,91,94].

Incidence risk, or information sufficient for its calculation, was reported in a few studies. Subgroup meta-analysis by MDI homology was performed using data from eight treatment-control comparisons [61,89,91,94] (Figure 4). There was no significant difference in the likelihood of becoming infected in MDI-positive piglets versus MDI-negative piglets regardless of MDI homology (RR = 1.0, 95% confidence interval of 0.95 to 1.05).

### 3.6. Outcome 2: Virus Titers–Standardized Mean Difference (SMD)

**Figure 5:**
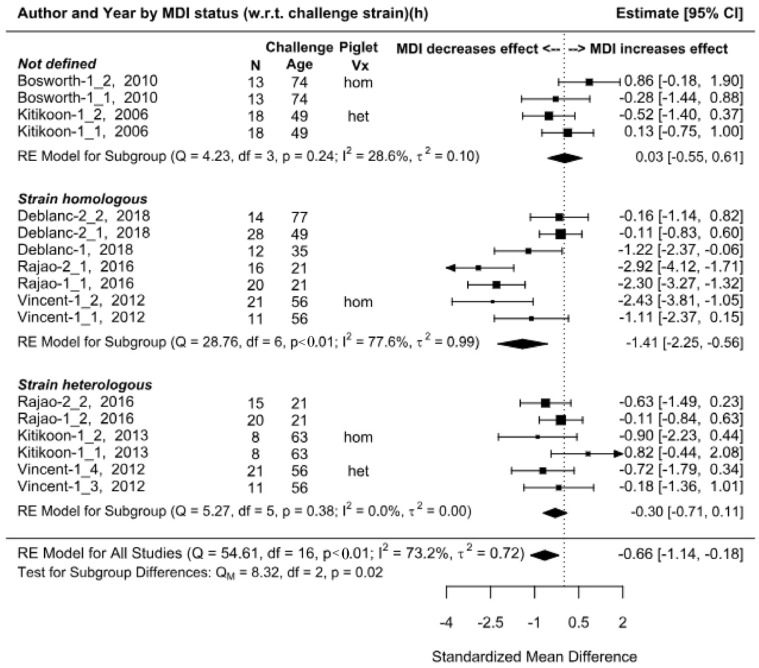
Sub-group meta-analysis forest plot of effects of IAV-S vaccine-induced MDI on virus titers in IAV-S challenged piglets: sub-grouping by sow vaccine homology with challenge virus. Composite effect sizes adjusted using estimate of high (h) correlation between repeated measures–comparisons with concurrently vaccinated offspring were included in the analysis. Study = MDI comparisons at the level of the trial and at the level of the treatment arm comparisons versus a control (in cases where trials include multi-arm comparisons); studies are label following the convention of [Author]-[Trial #]_[treatment arm comparison #], [year of publication]; for study citations refer to Table 1 as listed by author and year of publication.MDI = maternally derived immunity in offspring as induced through vaccination of dams against IAV-S. Meta-analysis sub-grouped by MDI status; Strain homologous = viral components of sow vaccine match the strain of the challenge virus; Strain heterologous = IAV-s virus antigenic components of the sow vaccine differ at the strain level from the challenge virus; Not defined = the IAV-S antigenic components of the maternal vaccine were not defined. *n*= total number of piglets in each treatment-control comparison; Challenge age is piglet days of age at challenge. Treatment-control comparisons involving concurrent IAV-S vaccination of piglets were included in meta-analysis; match of piglet vaccine stain with the challenge virus identified accordingly under the column heading Piglet Vx as hom (homologous) or Het (heterologous). Effect is mean virus titer (measured from nasal swab samples using virus isolation methods in all studies except for Bosworth et al. –virus quantified by PCR). Effect size is Hedges’ g, (standardized mean difference corrected for small sample size bias) calculated as a composite of effect sizes derived by collapsing first across homologous treatment arms and then across repeated time points, with pooled variances adjusted to account for assumed high (h) correlation of measures (0.75) from time point to time point. Columns on the right under the heading of Estimate are values and their 95% confidence intervals for the corresponding effect sizes and summary effect sizes. Effect sizes are represented by squares with size proportional to their weighted contribution to the summary effect size. Summary effects sizes are represented by diamonds. The dotted vertical line indicates a standardized mean difference of 0 (no effect difference between MDI-positive and MDI-negative groups) and points to the right of the line indicate MDI increases mean virus titers in offspring. I^2^ 95% uncertainty interval (lower bound, upper bound) = (51.21, 89.35). Refs [69,85,86,87,88,89].

Virus detection from nasal swab samples was reported in 10 publications. Data were sufficiently reported to extract or to calculate effect sizes from six publications [69,85,86,87,88,89] with all virus titer data extracted from graphs. The virus was quantified using virus isolation with group effects (mean titers) expressed in units of log10 TCID50/mL with the exception of a single study [85] where titers were derived using methods of quantitative PCR with units expressed as RT-PCR Log10. The effect was the mean virus titer as measured from nasal swab samples collected post-challenge, and the effect size was the difference in mean titers from treatment pigs (i.e., MDI-positive) versus control pigs (MDI-negative). The effect size is less than 0 when the mean virus titer for MDI-positive pigs is less than the mean titers for MDI-negative pigs, meaning that piglets with MDI shed less virus in upper respiratory passages than piglets without MDI. Differences in summary effect measures attributed to correlation adjustments between repeated measures over time were negligible (0.02 SMD) (See Figure 5 and Figure S1 (high (h) correlation), S2 and S3 (low correlation (l)). Overall, piglets with MDI shed less virus than piglets without MDI (standardized mean difference (Hedges’ g) = −0.66 (95% CI −1.14, −0.18) with the effect influenced most by MDI homology (Figure 5); piglets with homologous MDI had significantly reduced virus titers versus MDI-negative pigs, almost 1.5 standard deviation less which is interpreted as a large difference (g= −1.41, 95% CI: −2.25, −0.56). Where sow vaccines were undefined, there was no significant difference in virus titers between MDI-positive and negative groups. A slight reduction in virus titers in piglets with heterologous MDI versus controls was observed but the difference was not significant (g= −0.30, 95% CI: −0.71, 0.11). 

The removal of five effect sizes for comparisons involving concurrent IAV-S vaccination of piglets had minimal impact on subgroup summary effect measures (see Figure S1); the random effects model for all studies increased by 0.01 standardized mean difference (Hedges’ g), the confidence interval widened slightly (−0.65, 95% CI: −1.22, −0.08), and I^2^ increased negligibly (75.0%). However, results should be interpreted cautiously as studies were small, and few in number, and piglet vaccine timing, frequency (e.g., one vs. two doses), and platform type (e.g., WIV, LAIV, RP) were not considered in the analysis.

### 3.7. Outcome 3: Virus Shedding

Individual piglet data for measures of virus shedding from repeated nasal swab samples collected at multiple time points were available from three publications [11,61,89].

#### 3.7.1. Outcome 3a: Log Hazard Ratios (Log HR)–Beginning to Shed Virus Post-Challenge

**Figure 6:**
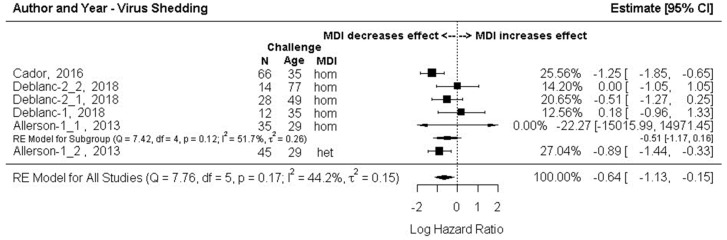
Subgroup meta-analysis forest plot of effects of IAV-S vaccine-induced MDI on likelihood (Log HR) of IAV-S challenged offspring to begin shedding virus: sub-grouping by sow vaccine antigenic homology with challenge virus. Study = MDI comparisons at the level of the trial and at the level of the treatment arm compari-sons versus a control (in cases where trials include multi-arm comparisons); studies are label following the convention of [Author]-[Trial #]_[treatment arm comparison #], [year of publica-tion]; for study citations refer to Table 1 as listed by author and year of publication.MDI = maternally derived immunity in offspring as induced through IAV-S vaccination of dams. Meta-analysis sub-grouped by MDI status; Strain homologous (hom) = viral components of sow vaccine match the challenge virus strain; Strain heterologous (het)= IAV-S virus antigenic components of the sow vaccine differ at the strain level from the challenge virus; effect is the hazard (the instantaneous rate of a first detection of virus in nasal swabs at any point in time during the post-challenge study period); effect size is the log of the hazard ratio (HR) between treatment and control groups as derived from Cox proportional hazard analysis; the dotted vertical line at 0 indicates the point of no difference in effects between the treatment and control groups; points to the right of the line indicate MDI increases the effect in the treatment group; I^2^ 95% uncertainty interval (lower bound, upper bound) = (0.00, 89.08); effect sizes are represented by squares with size proportional to their weighted contribution to the summary effect measure; values in the columns on the right hand side equal the weighted contribution is shown as a %, effect sizes and the summary effect sizes under the header of Estimate with their corresponding 95% confidence intervals [95% CI]; summary effects sizes are represented by diamonds [61,89,91].

The results of the subgroup meta-analysis, grouped by MDI homology, are shown in Figure 6. The summary effects size was significant (Log HR −0.64, 95% CI: −1.13, −0.15], and then back-transformed (HR (e^−0.64^) = 0.528) meant that the risk of piglets with MDI becoming infected was 47% lower on any given day during the study period versus piglets without MDI. An I^2^ of 44.2% suggests moderate heterogeneity but the 95% CI for the I^2^ estimate was wide (0.00, 89.08). The effect size for the homologous MDI treatment comparison labeled in Figure 6 as Allerson 2013.2 (Log HR −22.3, 95% CI: −15016, 14971) was considered an outlier with an extremely wide confidence interval. It involved infection via seeder pigs of a single animal (*n* = 1/20) that also continued to shed the virus for the duration of the study period [61]. This finding differed from all pigs in all other comparisons where all challenged animals became infected at some point post-challenge. This effect size was, however, weighted as 0% in the meta-analysis and had no impact on the overall or on sub-group summary effect sizes. The summary effect size for the homologous MDI subgroup was not significant (log HR = −0.51, 95% CI: −1.17, 0.16). A single sub-group comparison of heterologous MDI piglets (labeled in Figure 6 as Allerson 2013.1) showed a 70% reduced risk of shedding virus on any given day during the study period versus MDI-negative piglets. This finding should also be interpreted with caution as the study size was small (*n* = 45) [61].

#### 3.7.2. Outcome 3b: Days to Begin Shedding Virus Post-Challenge–Mean Difference (MD)

**Figure 7:**
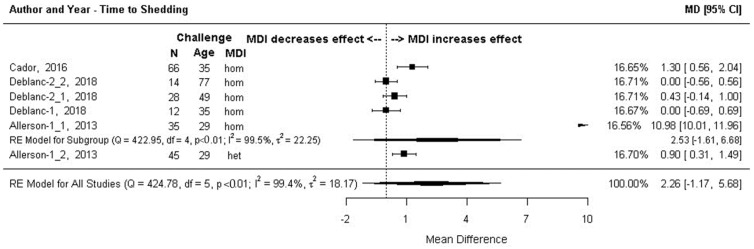
Subgroup meta-analysis forest plot of effects of IAV-S vaccine-induced MDI on difference in mean time (days) for IAV-S challenged offspring to begin shedding virus: sub-grouping by sow vaccine homology with challenge virus. Study = MDI comparisons at the level of the trial and at the level of the treatment arm com-pari-sons versus a control (in cases where trials include multi-arm comparisons); studies are la-bel following the convention of [Author]-[Trial #]_[treatment arm comparison #], [year of pub-lica-tion]; for study citations refer to Table 1 as listed by author and year of publication. MDI = maternally derived immunity in offspring as induced through IAV-S vaccination of dams. Meta-analysis sub-grouped by MDI status; Strain homologous (hom) = viral components of sow vaccine match the challenge virus strain; Strain heterologous (het)= IAV-S virus antigenic components of the sow vaccine differ at the strain level from the challenge virus; effect is mean time (days) for detection of virus in nasal swabs post-challenge; effect size is differences in mean time of MDI-positive vs. MDI-negative pigs to start shedding; the dotted vertical line indicates a mean difference of 0 (no difference between groups), points to the right indicates pigs with MDI take longer to begin shedding virus. I^2^ 95% uncertainty interval (lower bound, upper bound) = (98.41, 99.90); effect sizes are represented by squares with size proportional to their weighted contribution to the summary effect size; values in the columns on the right hand side equal the weighted contribution is shown as a %, effect sizes and the summary effect sizes under the header of Estimate with their corresponding 95% confidence intervals [95% CI]; summary effects sizes are represented by diamonds [61,89,91].

The effect was the mean time (days) for detection of the virus in nasal swabs collected post-challenge. The effect size was the difference in the mean time of MDI-positive versus MDI-negative pigs to start shedding. Subgroup meta-analysis, grouped by MDI homology, is shown in Figure 7. There was no statistical difference in the number of days to begin shedding overall (MD = 2.26 days, 95% CI: −1.17, 5.68), or at the homologous MDI subgroup level (MD = 2.53 days, 95% CI:−1.61, 6.68). The effect size for the single heterologous MDI comparison [61] (labeled in Figure 7 as Allerson 2013.1) was positive and significant (MD= 0.9, 95% CI: 0.31, 1.49) meaning that pigs with heterologous MDI took almost a day longer to begin shedding virus post-challenge than MDI-negative pigs.

**Figure 8:**
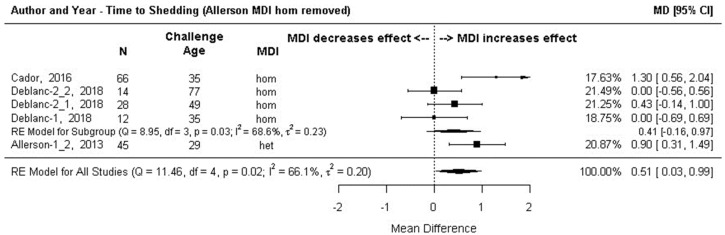
Subgroup meta-analysis forest plot of effects of IAV-S vaccine-induced MDI on the mean difference in time (days) for IAV-S challenged offspring to begin shedding virus–removing a single outlier effect size: sub-grouping by sow vaccine homology with challenge virus. Study = MDI comparisons at the level of the trial and at the level of the treatment arm com-pari-sons versus a control (in cases where trials include multi-arm comparisons); studies are la-bel following the convention of [Author]-[Trial #]_[treatment arm comparison #], [year of pub-lica-tion]; for study citations refer to Table 1 as listed by author and year of publication. MDI = maternally derived immunity in offspring as induced through IAV-S vaccination of dams. Meta-analysis sub-grouped by MDI status; Strain homologous (hom) = viral components of sow vaccine match the challenge virus strain; Strain heterologous (het)= IAV-S virus antigenic components of the sow vaccine differ at the strain level from the challenge virus; effect is mean time (days) for detection of virus in nasal swabs post-challenge; effect size is differences in mean time of MDI-positive vs. MDI-negative pigs to start shedding; the dotted vertical line indicates a mean difference of 0 (no difference between groups), points to the right indicates pigs with MDI take longer to begin shedding virus; a single study with an effect size on the extreme right of the plot was removed from the analysis (Allerson et al., 2013.2 MDI hom) effect size comparing the MDI homologous piglets (MD = 10.98, 95% CI: 10.01–11.96); I^2^ 95% uncertainty interval (lower bound, upper bound) = (98.41, 99.90); effect sizes are represented by squares with size proportional to their weighted contribution to the summary effect size; values in the columns on the right-hand side equal the weighted contribution is shown as a %, effect sizes and the summary effect sizes under the header of Estimate with their corresponding 95% confidence intervals [95% CI]; summary effects sizes are represented by diamonds [61,89,91].

The I^2^ estimate indicated high heterogeneity. A single outlying homologous MDI effect size, labeled in Figure 7 as Allerson 2013.2, (MD = 10.98 days, 95% CI: 10.01, 11.96) was removed from the analysis resulting in an overall summary effect size that indicated piglets with MDI take approximately a half day longer to begin shedding virus versus piglets without MDI (Figure 8). The homologous MDI subgroup summary effect size indicates no significant difference in effects between MDI-positive and MDI-negative pigs (MD = 0.51 days, 95% CI:−0.16, 0.97), I^2^ estimate suggested moderate (I^2^ = 66.1%, 95% CI: 2.99–96.25).

#### 3.7.3. Outcome 3c: Log Hazard Ratios (Log HR) for Ceasing to Shed Virus

**Figure 9:**
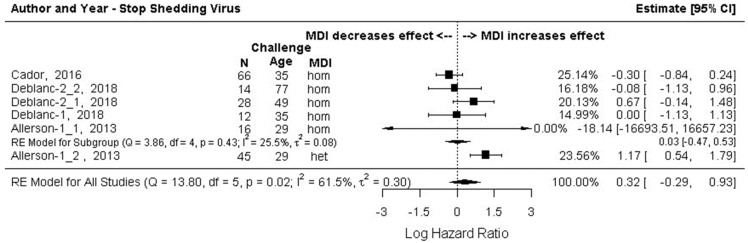
Subgroup meta-analysis forest plot of effects of IAV-S vaccine-induced MDI on the log hazard ratio (Log HR) of IAV-S challenged offspring for stopping virus shedding: sub-grouping by sow vaccine homology with challenge virus. Study = MDI comparisons at the level of the trial and at the level of the treatment arm com-pari-sons versus a control (in cases where trials include multi-arm comparisons); studies are la-bel following the convention of [Author]-[Trial #]_[treatment arm comparison #], [year of pub-lica-tion]; for study citations refer to Table 1 as listed by author and year of publication. MDI = maternally derived immunity in offspring as induced through IAV-S vaccination of dams. Meta-analysis sub-grouped by MDI status; Strain homologous (hom) = viral components of sow vaccine match the challenge virus strain; Strain heterologous (het)= IAV-S virus antigenic components of the sow vaccine differ at the strain level from the challenge virus; effect is the hazard (the instantaneous rate in virus positive piglets of ceasing to shed virus at any point during the post-challenge study period); effect size is the log of the hazard ratio (HR) as derived from cox proportional hazard analysis; the dotted vertical line at 0 indicates the point of no difference (log HR = 0) and points to the right of the line indicate MDI increases the effect in the treatment group; I^2^ 95% uncertainty interval (lower bound, upper bound) = (3.64, 89.69); effect sizes are represented by squares with size proportional to their weighted contribution to the summary effect measure; values in the columns on the right hand side equal the weighted contribution is shown as a %, effect sizes and the summary effect sizes under the header of Estimate with their corresponding 95% confidence intervals [95% CI]; summary effects sizes are represented by diamonds [61,89,91].

Data from the six comparisons in Section 3a were assessed for the likelihood of stopping viral shedding where the hazard was the likelihood of infected pigs ceasing to shed virus on any given day during the study period. Effect size was the difference in the hazard for the MDI-positive group versus the MDI-negative group (Log HR). Subgroup meta-analysis results (sub-grouped by sow vaccine homology) are shown as a forest plot in Figure 9. Infected pigs with or without MDI had the same likelihood of ceasing to shed the virus on any given day during the study period. The confidence interval of the summary effect size (log HR = 0.32) included the point of no difference (−0.29, 0.93). The summary effect measures for the homologous MDI subgroup were also not significant. However, the effect size from the single heterologous MDI comparison (Allerson 2013.1, Figure 9) was significant with a log HR of 1.17 (95% CI: 0.54, 1.79) indicating piglets in this study with heterologous MDI were over three times as likely (HR = 3.22) to stop shedding virus on any given day during the study period versus piglets without MDI. A single effect size for the homologous MDI comparison discussed above [61] (Allerson 2013.2, Figure 9) had an extremely wide confidence interval. This effect size was weighted as 0% in the meta-analysis and so had no impact on the summary effect size.

#### 3.7.4. Outcome 3d: Post-Infection Days to Cease Shedding Virus–Mean Difference (MD)

**Figure 10:**
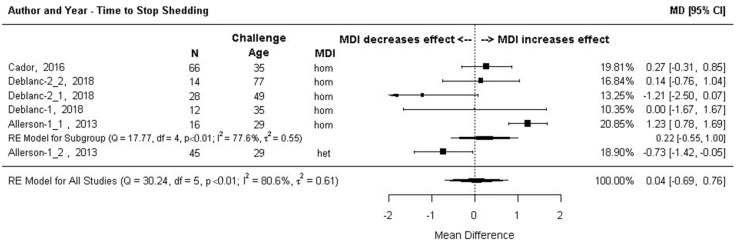
Subgroup meta-analysis forest plot of effects of IAV-S vaccine-induced MDI on the mean difference (days) in time for infected IAV-S challenged offspring to stop shedding virus: sub-grouping by sow vaccine homology with challenge virus. Study = MDI comparisons at the level of the trial and at the level of the treatment arm com-pari-sons versus a control (in cases where trials include multi-arm comparisons); studies are la-bel following the convention of [Author]-[Trial #]_[treatment arm comparison #], [year of pub-lica-tion]; for study citations refer to Table 1 as listed by author and year of publication. MDI = maternally derived immunity in offspring as induced through IAV-S vaccination of dams. Meta-analysis sub-grouped by MDI status; Strain homologous (hom) = viral components of sow vaccine match the challenge virus strain; Strain heterologous (het)= IAV-S virus antigenic components of the sow vaccine differ at the strain level from the challenge virus; effect is mean number of days virus is detected in nasal swabs of infected piglets; effect size is difference in the mean days MDI-positive pigs shed virus versus MDI-negative pigs; the dotted line indicates a mean difference of 0 (no difference between MDI-positive and MDI-negative groups in mean days shedding); points to the right of the line indicate that pigs with MDI shed virus longer; I^2^ 95% uncertainty interval (lower bound, upper bound) = (47.40 96.61); effect sizes are represented by squares with size proportional to their weighted contribution to the summary effect measure; values in the columns on the right hand side equal the weighted contribution is shown as a %, effect sizes and the summary effect sizes under the header of Estimate with their corresponding 95% confidence intervals [95% CI]; summary effects sizes are represented by diamonds [61,89,91].

Data from the six comparisons in Section 3b were assessed for mean difference (MD) in time for infected piglets to stop shedding. Subgroup meta-analysis results are shown as a forest plot in Figure 10. There was no significant difference between MDI-positive and MDI-negative piglets in the number of days infected piglets shed virus overall (MD = 0.04, 95% CI:−0.69, 0.76), or for the homologous MDI subgroup (MD = 0.22, 95% CI:−0.55, 1.00). The single heterologous MDI effect size (Allerson 3013.1) was significant indicating piglets with heterologous MDI shed virus for almost ¾ of the day less than MDI-negative piglets (MD-0.73, 95% CI:−1.42, −0.05). As with the findings discussed above, the results from a single comparison should be interpreted with caution.

### 3.8. Indirect Measure of Protection–Correlate of Protection (CoP)

#### Outcome 4: Serum Hemagglutination Inhibition (HI) Titers (Qualitative Synthesis)

HI titers were reported in reference to challenge viruses in 11/15 studies and effects for all but one [61] were presented in a graphical format and required manual extraction of mean titer data from graphs. Study parameters contextually important for interpretation of HI titers are summarized in Table 1. Measures of dispersion for HI titer data were infrequently reported or could not be extracted. More than half of all studies (7 studies) [85,86,87,88,90,94] ended at 5 days or less post-challenge (Table 1, Figures S4 and S5); an insufficient period of time for detection of a serologic response to challenge [92,95,96]. Mean HI titers across all studies were jointly plotted for descriptive comparison of treatment and control arm titers as one of two stacked graphs depending on whether or not piglets were concurrently vaccinated; studies involving piglets that were not vaccinated are shown in the stacked graph in Figure S4 and studies involving piglets that were also vaccination are shown in Figure S5. A rise in mean HI titers post-challenge was not detected in any study where the baseline HI mean titers of MDI-positive piglet groups exceeded 1:40 at the time of challenge (Table 1, Figures S4 and S5). Additional explanation of observed HI titers is provided in Supplementary Materials (Text S5).

### 3.9. Direct Measures of Infection–Non-Specific Endpoints

#### VAERD

Measures of lung pathology for the diagnosis of VAERD was not, *a priori*, an outcome of interest in this review; however, VAERD was clinically reproduced and assessed as a stated objective in seven studies; therefore, descriptive findings are summarized narratively for the interested reader in Supplementary Materials Text S6.

### 3.10. Outcome 5: Mean Difference (MD) in Average Daily Gain

Piglet weights were extracted for calculation of an effect size from two studies [89]. Piglet weighs were reported in a third study but an effect size could not be calculated due to insufficient reporting [69]. There was a mean difference ADG of 180 g/day (0.18 kg/day) in the 48-h post-challenge period in favor of MDI-positive pigs versus MDI-negative pigs but this difference was not significant (95% CI: −30 g, 390 g) (Figure S5). An additional explanation of observed mean differences in ADG is provided in Supplementary Materials (Text S7). 

### 3.11. Outcome 6: Coughing (Insufficient Data for Meta-Analysis)

Coughing was measured in three publications (four studies) [69,88,94] but reported sufficiently to calculate the effect size (RR) in a single study (Kitikoon,2006) [69]. Coughing reported narratively in both studies in Rajao (2016) [88], was similar to the findings of Kitikoon (2006) [69], where moderate to severe coughing was observed only in piglets with strain heterologous MDI but not in pigs with strain homologous MDI, or in piglets from unvaccinated dams (i.e., MDI-negative). Piglets were not vaccinated in the Rajao studies. An additional explanation of observed mean differences in ADG is provided in the Supplementary Materials (Text S8). 

### 3.12. Risk of Bias across Studies

Publication bias was assessed for the meta-analysis of virus titers only as no other meta-analysis included 10 or more treatment-control comparisons and there was no evidence of small study effects.

### 3.13. Confidence in Cumulative Evidence

Confidence in cumulative evidence was partially assessed. Illustrative comparative risks are not meaningful constructs for challenge trial outcomes as effect sizes generally extrapolate poorly to field or ‘real world’ conditions. Therefore, confidence across all evidence was downgraded by a level of indirectness. Assessment of imprecision and interpretation of outcome differences was incomplete as minimal meaningful outcome differences with respect to appreciable benefit or harm (e.g., VAERD) have not been established in the field [14]. Publication bias was assessed for the meta-analysis of virus titers but not for others as too few studies were included in each. A partial evidence profile is available in Supplementary Materials (Table S7) and, posteriori, it was decided to omit overall ratings for GRADE quality of evidence, calculations of illustrative comparative risks, and the ‘Summary of Findings’ table. A summary of effect sizes and study size parameters for each of the outcomes are listed in Table 3.

## 4. Discussion

Overall, maternal immunity significantly reduced virus titers when piglets were challenged with homologous but not with heterologous virus strains. The impact of sow vaccination on the likelihood of infection is unclear and differed by the outcome measured. When measured as a risk ratio, there was no significant difference in the incidence risk (i.e., risk of becoming newly infected) between pigs with MDI and no MDI over the entire period of follow-up, regardless of MDI homology. However, when measured as a log hazard ratio, the hazard of infection on a daily basis was significantly reduced and the mean time between exposure and infection was significantly longer in pigs with MDI. Different conclusions about the likelihood of infection could be attributed to the type of underlying measure of risk used for evaluation but also, in this case, due to respective omissions in reported key data and the resulting differences in the subset of studies used in the meta-analysis of relative risks (RR) versus those used in meta-analyses of hazard ratios (HR) and of mean differences (MD). Once pigs were infected, there was overall, no difference between MDI-positive or negative pigs in the mean time to cease shedding virus, or when measured as a hazard ratio, of the instantaneous likelihood of ceasing to shed virus during the period of study. Very few studies reported the impacts of MDI on ADG or on the incidence of coughing. 

### 4.1. Selection and Measure of Outcomes

Influenza research now centers on the understanding of the relationship of epitope immunodominance with vaccine effectiveness through the evaluation of multiple outcomes and across multiple virus exposures [21,97]. Outcomes selected for study by researchers varied across studies and these differences limited the strength of findings in this review. In this systematic review, we selected six outcomes believed important and commonly reported across studies. To account for the full spectrum of immune responses when evaluating IAV-S vaccine protection, the consideration of multiple correlates of protection is important [98,99,100]. As such, the interpretation of indirect immunologic responses in this review was limited because none of the six outcomes was a correlate of cell-mediated immunity and because HI serum immunoglobulin titers (against challenge virus antigens only) but not ELISA assay titers were considered; both Rajao (2016) [88] and Sandbulte (2014) [94] showed ELISA assays were more sensitive for detecting non-neutralizing antibodies than the HI assay. Until such time agreement is reached within the IAV-S vaccine research community on priority outcomes to report across all studies, capture of findings from all relevant studies in systematic reviews and meta-analyses will likely be sub-optimal. In human healthcare, initiatives to establish Core Outcome Sets (COSs) as standards for the collection of an agreed minimum set of research trial outcomes have improved the availability of research evidence for multiple stakeholders [101]. This concept has been recently applied to veterinary research [102] and the establishment of COSs for IAV-S vaccine research in swine may improve the inclusion of all relevant studies and a wider swath of important outcomes in research syntheses.

### 4.2. Reporting of Key Data Required for Meta-Analysis

Reporting insufficiencies limited information that otherwise may have been gained. Most outcomes were reported as group-level effects and calculation of effect sizes for inclusion in the meta-analysis was necessary. Key data required for meta-analyses, such as total numbers of infected pigs in each group or measures of dispersion, were frequently lacking or difficult to extract (i.e., manual extrapolation from graphs). As a result, effect sizes could not be calculated for all eligible studies, and a substantial number of studies were omitted from meta-analyses. 

### 4.3. Contextual (Clinical) Heterogeneity

Quantifying variation allows the reader to know if the effect size is consistent or differs widely from study to study [73]. Differences in study features such as vaccine and challenge virus antigenic composition, dosing, and method of delivery, are all sources of inter-study variation and can influence outcomes [103,104]. Heterogeneity was moderate or greater in all meta-analyses. Across the 15 studies, study characteristics potentially contributing to contextual (clinical) heterogeneity (where there may be multiple ‘true effects’ depending on contextual variables) included diverse sow IAV-S exposure histories (Figure 2), a wide range of piglet ages at challenge (Table 1), diverse combinations of vaccine/challenge virus homology employed (Table 2, see also Tables S3 and S4), variable mean HI titers at the time of challenge (Table 1), concurrence of piglet IAV-S vaccination (Table 1), differences in piglet vaccine platforms and vaccination schedules (Table S4), and differences in the frequency and timing of sample collections and of assay choices (Table S2). MDI homology was the only subgroup variable investigated as a source of between-study heterogeneity as few studies grouped otherwise by these variables.

### 4.4. HI Titers: Widely Reported, Limited Information for Quantitative Synthesis

It is convenient to measure piglet HI titers as a means of evaluating the effectiveness of a maternal vaccination program to confer MDI. However, to identify an HI titer threshold in offspring at which vaccine-induced MDI is considered protective (or conversely suppressive), the titer must also correlate with a direct outcome measure (such as virus detection or clinical disease) at which protection from infection or disease is also demonstrated [28,29,105]. This correlation must then be consistent across multiple studies to be validated as a correlate of protection (CoP) [97,106]. A piglet’s baseline HI titer from vaccine-induced MDI is influenced by changes in the timing, antigenicity, and dosing used in the maternal pre-farrowing vaccine program. A 1:40 HI titer is accepted as protective in humans and animals [48,48]; however, the appropriateness of the extrapolation of this threshold titer as a protective level of vaccine-induced MDI in young pigs has not been established. The effect of differences in pre-challenge levels of MDI (reported as high or low HI titers) on piglet responses to challenge was investigated in a single study, where the baseline HI titer at challenge (e.g., high or low) correlated significantly with differences in piglet post-challenge serologic responses and with the amount and duration of virus shedding [89]. Protective (or conversely, immunosuppressive) threshold titers from vaccine-induced MDI need to be established to optimize decisions on sow vaccination program timing, dose frequency, and the antigenic and adjuvant composition of maternal vaccines. 

### 4.5. Piglet Baseline MDI

Maternal vaccination program effectiveness is a function of the volume and quality of colostrum each piglet receives where differences may contribute to both within and between-study variability. Using sub-group meta-analyses, we demonstrated that qualitative differences in MDI homology impacted outcomes. Differences in individual piglet baseline measures of MDI were investigated as a within-study variable in a single study [11]; authors reported piglets with low baseline levels of transferred immunity behaved more like MDI-negative pigs than high-level MDI groupmates. These findings highlight the importance of using individual piglet data versus aggregated group means. The attribution of variation in piglet baseline MDI to outcome measure dispersions may not be appreciated if using group mean titers for analyses. Across the body of evidence, treatment group baseline mean titers varied considerably from study to study (Table 1, Figures S4 and S5) but were not investigated further as a potential between-study source of heterogeneity. 

### 4.6. Gaps in Maternal Exposure History

Immunologic responses following vaccination differ from those following infection but little is known about how the type of initial exposure (vaccine or wild-type virus) impacts immunologic imprinting [21,107,108,109,110]. The nature of anti-IAV immunoglobulins in colostrum is determined by the sow’s total and sequential order of IAV-S exposures (i.e., vaccines and natural viruses), where the first exposure shapes all subsequent immunologic responses. This immune phenomenon is referred to as ‘original antigenic sin’ (OAS), immunologic superiority, and most recently, immune imprinting [59,108,111,112,113]. The impacts of imprinting upon responses to a second heterologous exposure may be consequential and at the expense of de novo production of neutralizing antibodies against the second virus [21,23]. Therefore, breeding herd exposure history at the individual animal level is an important consideration when evaluating maternal IAV-S vaccine program effectiveness. In this review, reporting of dam exposure histories and antigenic characterization of vaccines was limited or absent in almost a third of the studies. This diminished potential data available for sub-group analyses by MDI homology and was problematic for making inferences from a third of the studies in this review.

### 4.7. MDI and Response to Secondary Exposure

We identified a single publication (two studies) investigating the impact of MDA on a secondary (or subsequent) exposure [89]. In both studies, the secondary challenge was conducted using a virus strain identical to that used as the primary challenge. As no animals in either the MDA positive or negative groups were infected following the second challenge, the assessment of the impact of MDA on responses to secondary exposure was limited. How animals respond to subsequent challenges is particularly relevant to large commercial production situations where piglets are likely to encounter first exposures in the farrowing barns, and then be exposed again to an existing or to new circulating IAV-S viruses during the grower-finisher stages of production, particularly in cases of multi-sourced housing [38,103]. The impacts of MDI on the secondary heterologous challenge have yet to be investigated and are, therefore, warranted. 

### 4.8. VAERD and Influenza in Pigs

Vaccine-associated disease is an important consideration in decisions on vaccine platforms [30,114]. A severe and distinct immunopathology of the lungs, vaccine-associated enhanced respiratory disease (VAERD) [115], is induced in pigs under experimental conditions in association with influenza vaccines [13,35,92,115,116,117]. VAERD has also been induced in unvaccinated but MDA-positive offspring from dams previously vaccinated with a WIV [92] complicating further attribution of VAERD to vaccination of the dams versus vaccination of piglets. Although the enhanced disease has been reproduced experimentally in ferrets [34], and been associated with post-marketing use of licensed inactivated influenza vaccine preparations in human populations [32,33], the practical risks of VAERD in commercial swine settings associated with either passive (i.e., MDI) or vaccine-induced immunity are unknown [117]. 

### 4.9. MDI and Immunosuppression

Over forty years ago maternal antibodies were shown to cause vaccine failure [17,18,19] and passive IgG is an established immunosuppressant [1,16]. Vono et al. (2019) [18] showed in mice that the degree of immunosuppressive effects of MDI was determined by the antibody/antigen ratio. High levels of maternal antibodies drastically inhibited immunological maturation of responses needed for immune memory and long-term protection, and reduced responses to a second antigenic exposure. Conversely, inhibition was partially abrogated with low or intermediate levels of maternal antibodies, or with the use of high antigen doses in offspring vaccines [18]. Vono et al.’s findings are also consistent with those made in pigs decades earlier by Renshaw [118]. Renshaw’s work was one of three MDI and influenza studies in swine that demonstrated MDI as immunosuppressive in pigs [92,118,119]. Although these studies did not meet our inclusion criteria for this review, each contributed observation highlighted an important area for future pre-farrowing IAV-S vaccine research with respect to decisions on the timing, dose, and antigenic composition of maternal vaccines in swine. Three strategies have been proposed to mitigate immunosuppression in infants caused by human maternal vaccinations [18] where each is potentially applicable to future research on swine vaccines used pre-farrowing; (1) booster young piglets with high dose novel antigens during periods of waning maternal immunity, (2) use distinct antigenic vaccines in dams versus piglets to minimize inhibition of piglet B cell maturation to important immunodominant epitopes, (3) restrict use of adjuvanted vaccines in dams such that induced MDA titers do not exceed minimal titers needed for protection against clinical disease in piglets. 

### 4.10. Gaps in IAV-S Maternal Vaccine Research

The impact of the sow exposure history on the nature and quality of MDI for the protection of offspring has not been investigated in a controlled challenge setting, nor has the attribution of MDI to undesirable effects such as immunosuppression or VEARD been assessed in the field. 

Insight from this systematic review and meta-analyses was limited owing, in large part, to three concerns; inconsistent researcher selection of outcomes across studies, contextual heterogeneity, and inconsistent reporting of the key data required for meta-analysis. Comprehensive characterization of all IAV exposure (i.e., vaccine, natural, and challenge), and consistent reporting of measures of centrality, dispersion, and treatment group numerators and denominators, are necessary for the inclusion of research findings in systematic reviews and meta-analyses. Adherence to reporting guidelines such as REFLECT [120], and establishing core outcome sets (COSs) [101,102] for influenza vaccine research in swine species is encouraged. As the evaluation of responses to vaccines evolves to include correlates of protection (CoPs) for cell-mediated immunity, swine health researchers, as members of the One Health community of practice, will be challenged to expand and to consistently measure and report the most informative outcomes for influenza vaccine research [121,122,123,124]. 

In the field, individual animal exposure to more than one sub-type and/or strain of influenza virus is common [39,125,126,127] yet none of the challenge trials included in this review involved challenging piglets with more than one virus strain. Two studies involved challenging piglets a second time (secondary exposure) [89] but in both studies, the same strain of virus was used for both the primary and the secondary challenge. Further research is warranted to investigate how the timing and frequency of pre-farrowing vaccination, the antigenic composition of maternal vaccines (e.g., heterologous prime-boost vaccination strategies), and how the use of non-WIV vaccine platforms in dams (of which none are currently licensed for use in pregnant dams) impact piglet responses to primary, and then to secondary challenges, and piglet responses to multi-valent (i.e., more than one sub-type, and/or strain) secondary challenges [47].

Lastly, owing to the complexity of the virus-host relationship, research to establish threshold levels of vaccine-induced MDA (where the benefits of passive transfer outweigh disadvantages) will be complex to design and likely necessitate measuring of multiple immunologic outcomes, require analysis of individual pig data, and be conducted under conditions were the kinetics of virus exposure more closely mimics viral transmission in large populations. Given that commercial populations differ substantially in size and viral dynamics versus challenge trial populations, field trials will ultimately be important for determining the effectiveness of sow vaccination to protect offspring against IAV-S [128].

## 5. Limitations

### 5.1. Limited External Validity

Challenge trials provide essential immunologic insights otherwise difficult to derive from field trials and hence their importance for licensing of influenza vaccines [129,130,131] but they also lack external validity [132,133,134,135,136,137]. All studies in this review were single strain challenges and investigation of piglet responses to a second influenza challenge was conducted in two studies. Additionally, study populations were small in comparison to commercial populations, each with differing immunologic competencies, and where exposure to IAV-S is dynamic and highly variable [14,38,39].

### 5.2. Exclusion of Control Groups with MDI-negative but Vaccinated Piglets

Although the efficacy of vaccines administered to the piglet was investigated in nine studies included in this review [69,85,86,87,90,93,94], the evaluation of piglet vaccine efficacy was not the review objective, and therefore, all treatment arms, with and without vaccinated piglets, were compared only to MDI-negative controls groups where none of the control piglets had been vaccinated against IAV-S. However, the exclusion of control groups with MDI-negative but vaccinated piglets meant control arm totals were diluted across multiple treatment arms and contributed to increased effect size uncertainty.

### 5.3. Single Reviewer Data Extraction and Risk of Bias Assessment

Single-reviewer data extraction and assessment of bias may have increased the risk of errors in extracted measures and in the interpretation of bias. 

## 6. Conclusions

Our findings suggest upper respiratory virus titers are reduced in piglets with vaccine-induced homologous MDI, but not for piglets with heterologous MDI. The clinical importance of reduced virus titers in a commercial farm setting is unknown considering also that there was no difference in the relative risk of infection. With the exception of reported virus titer data, data from a few studies were included in meta-analyses of incidence risk of infection, duration of virus shedding, coughing, average daily gain, or HI titers. Differences in researcher choices of outcomes to measure, and omissions and deficiencies in reporting of outcome further limited the number of studies that were included in meta-analyses.

Challenge trials investigating the impacts of vaccine-induced MDI on piglet responses to a secondary homologous or heterologous challenge were rare or absent, respectively. Additional research is also needed to understand how choices for the clinical design of trials impact outcomes. This includes decisions on concurrent vaccination of piglets, the piglet’s age at first challenge, the frequency, number, and order of exposure of challenge strains, and the timing, dose and epitope homology of field strains versus the sow vaccine strain(s). Overall, the body of available challenge trial evidence did not support or refute the IAV-S vaccination of sows as protective for offspring. 

## Figures and Tables

**Table 1:** Study design features and HI titers of MDI-positive piglets at challenge for 16 IAV-S vaccine-induced MDI challenge trials published from 1990 to 2021.

			(Days of Age)				
Study-Trial # Year	Affiliation	Study Length (dpc)	1st Chal	2nd Chal	Piglet vx ^₮^	Effect ^Ŧ^ no pg vx (GMT ^ŦŦ^ at Chal)	Effect ^Ŧ^ with pg vx (GMT ^ŦŦ^ at Chal)
Rajao-1 2016 [88]	ARS/USDA	17	**21 ^‡^**			**1:485 (hom)**	
Rajao-2 2016 [88]	ARS/USDA	17	**21 ^‡^**			**1:787 (hom)**	
Allerson 2013 [61]	UMinn	14	**28.5 ^‡^**			**1:320 (hom)**, 1:17 (het)	
Cador 2016 [91]	ANSES	28	**35 ^‡^**				
Genzow-1 2018 [93]	BIAH	5	**35**		(5)		
*Pyo 2015 [83]	VIDO	5	**37**		(16,30)		
Genzow-2 2018 [93]	BIAH	5	**42**		(5)		
DeBlanc-1 2018 [89]	ANSES	42	**35**	**63**		**~1:597 (hom)**, **1:65 (hom)**	
Kitikoon 2006 [69]	ISU	21	49		(21,35)	1:11 (nd)	1:14 (nd_het)
Loving 2014 [90]	ARS/USDA	5	49		(21,35)	1:25 (hom), <1:10 (het)	1:26 (hom_hom), <1:10 (het_hom)
DeBlanc-2 2018 [89]	ANSES	48	49			~1:28 (hom)	
DeBlanc-2 2018 [89]	ANSES	48	**49**	77		**~1:49 (hom)**, <1:10 (hom)	
Sandbulte 2014 [94]	ARS/USDA	8	52.5		(18,32)	<1:10 (het)	<1:10 (het_het), <1:10 (het_het_LAIV), <1:10 (het_het_LAIVx2)
Vincent 2012 [86]	ARS/USDA	5	56		(14,28)	~1:16 (hom), <1:10 (het)	~1:12 (hom_hom), ~1:14 (hom_hom_LAIV), <1:10 (het_het), <1:10 (het_het_LAIV)
Kitikoon 2013 [87]	ARS/USDA	5	**63**		(28,49)	1:25 (hom)	**1:80 (het_hom)**
Genzow-3 2018 [93]	BIAH	5	**70**		(5)		
Bosworth 2010 [85]	ISU	4	74		(21,35)	1:16 (nd)	<1:10 (nd_hom), 1:17 (nd_hom_RP)
DeBlanc-2 2018 [89]	ANSES	21	77			<1:10 (hom)	

MDI = IAV-S vaccine-induced maternally derived immunity; all treatment (intervention) arms were MDI positive; all control arms were MDI negative; dpc = days post-challenge; y = yes, values were sufficiently reported; *n*= no, values were not sufficiently reported; ^₮^ pgvx = piglet days of age at vaccination (for studies with concurrently IAV-S vaccinated offspring); aham is ^Ŧ^ phenotypic match of vaccine hemagglutinin (HA) antigenic composition, by sub-type, and by strain; GMT = geometric mean hemagglutination inhibition (HI) titer for groups of piglets in MDI-positive treatment arms (as sampled closest to challenge day) with values above 1:40 in **bold** (all GMTs for MDI-negative arms were 1:10 or less and not shown); information on vaccine homology in reference to challenge viruses for each treatment group is shown in brackets () where hom = vaccine antigenically homologous with challenge virus; het = vaccine heterologous to challenge virus; nd= vaccine antigenicity not-defined; LAIV= piglet vaccine type was live attenuated influenza vaccine; RP= piglet vaccine type was a replicon particle vaccine) with sow vaccine listed first followed, where applicable, by piglet vaccine and then piglet vaccine type, each separated by an underscore _ (i.e., maternal vaccine homology_piglet vaccine homology_piglet vaccine type); ^‡^ offspring exposure also via indirect contact and/or via seeder pigs in direct and/or indirect contact; cells shaded in grey, gold, or orange indicates studies funded by governmental organizations in the USA, France, and Canada, respectively; * Pyo et al. [83] was included in descriptive totals but no outcome data were extracted.

**Table 2:** The frequency of the combinations applied of the 20 possible combinations of antigenic matches (of maternal and piglet vaccines to the challenge virus) across each of the study arms (*n*= 45) employed in 16 MDI challenge trials.

Population IAV-S Vaccinated			Dam Vaccine Match ^Ŧ^		Piglet Vaccine Match ^Ŧ^			Combination Frequency (No.)		
No.	Dams Vaccinated	Piglets Vaccinated	Subtype	Strain	Subtype	Strain	Arm Designation	Studies	Study Arms	Offspring
1	yes	no	het	N/A	N/A	N/A	MDI+	none	-	-
2	yes	no	hom	hom	N/A	N/A	MDI+	8	11	131
3	yes	no	hom	het	N/A	N/A	MDI+	8	9	103
4	yes	no	not defined		N/A	N/A	MDI+	5	5	135
5	yes	yes	not defined		het	N/A	MDI+	none	-	-
6	yes	yes	not defined		hom	hom	MDI+	1	2	20
7	yes	yes	not defined		hom	het	MDI+	4	4	102
8	yes	yes	hom	hom	het	N/A	MDI+	none	-	-
9	yes	yes	hom	hom	hom	hom	MDI+	2	4	31
10	yes	yes	hom	hom	hom	het	MDI+	none	-	-
11	yes	yes	hom	het	het	N/A	MDI+	none	-	-
12	yes	yes	hom	het	hom	hom	MDI+	2	3	18
13	yes	yes	hom	het	hom	het	MDI+	3	7	54
14	yes	yes	het	N/A	het	N/A	MDI+	none	-	-
15	yes	yes	het	N/A	hom	hom	MDI+	none	-	-
16	yes	yes	het	N/A	hom	het	MDI+	none	-	-
17	no	no	N/A	N/A	N/A	N/A	MDI-ve Control arm	12	22	266
18	no	yes	N/A	N/A	het	N/A	Excluded ^₮^	none	-	-
19	no	yes	N/A	N/A	hom	hom	Excluded ^₮^	5	7	49
20	no	yes	N/A	N/A	hom	het	Excluded ^₮^	7	10	93

Not all combinations were applied and application of more than one vaccine and/or more than one challenge virus was possible in multi-armed studies. No. = combination number (1 of 20 different possible combinations of the three antigenic variables; maternal vaccine, piglet vaccine (s), and challenge viruses); MDI = IAV-S vaccine-induced maternally-derived immunity; MDI+ = offspring of vaccinated dams; MDI-ve = offspring of unvaccinated IAV-S negative dams (i.e., control dams); ^Ŧ^ phenotypic match of vaccine hemagglutinin (HA) antigenic composition, by sub-type, and by strain, in reference to the challenge virus where hom = homologous, het = heterologous, not defined = no antigen information reported, and N/A = not applicable; ^₮^ study arms involving combination No. 18, 19, or 20 were excluded from analyses (i.e., IAV-S negative and unvaccinated dams but IAV-S vaccinated offspring).

**Table 3:** Highlights of meta-analyses of six outcomes of IAV-S vaccine-induced MDI challenge trials in pigs.

Outcomes	Summary Effect Size (95% CI)	No. of Offspring (Studies)	Comments
Direct measures of infection (nasal swab samples, detection by PCR or virus isolation):			
1. Virus detection (incidence)	RR 1.0 (0.95–1.05) I^2^ = 49.5% (95% CI 0.0, 77.5)	**240 (5)**	Piglets with MDI were not more likely to be infected than piglets without MDI
2. Virus titers	SMD = −0.66 (95% CI −1.14, −0.18) I^2^ = 73.2% (95% CI 51.21–89.35)	**267 (8)**	Piglets with homologous MDI had 1.5 standard deviations less virus detected vs. piglets without MDI
3. Virus shedding:			
(i) Detection of shedding	Log HR −0.64 (−1.13, −0.15) I^2^ = 44.2% (95% CI 0.00–89.08)	200 (4)	Instantaneous infection risk was 47% lower for MDI+’ve versus MDI–‘ve piglets
(ii) Days to begin shedding	MD = 2.26 (95% CI −1.17, 5.68) I^2^ = 99.4% (95% CI 98.4, 99.9)	200 (4)	No difference between groups in no. of days to begin shedding post-challenge.
(iii) Ceasing to shed	Log HR 0.32 (−0.29, 0.93) I^2^ = 61.5% (95% CI 3.64, 89.69)	181 (4)	No real difference in the likelihood of piglets from either group to cease shedding during the study period.
(iv) Days of shedding	MD = 0.04 (95% CI −0.69, 0.76) I^2^ = 80.6% (95% CI 47.4, 96.6)	181 (4)	No difference between groups in the no. of days infected piglets shed virus.
**Indirect measures and clinical signs:**			
4. HI titer		40 (2)	No summary effect measure calculated (descriptive only)
5. Avg. daily gain (at 48 hrs pc)	MD = 0.18 (95% CI −0.03, 0.39) I^2^ = 82.2% (95% CI 44.43, 98.47	54 (2)	No difference in weight gain between MDI+ and MDI- groups.)
6. Coughing		48 (1)	Piglets with MDI were 2.3 times as likely to cough vs. piglets without. No summary effect measure (single study only)

Population: Vaccinated dams and their piglet offspring; Intervention: IAV-S vaccination of dams pre-farrowing; Comparison: IAV-S negative offspring from dams with no prior exposure to IAV-S; Study type: challenge trials.

## Data Availability

The authors confirm that all data underlying the findings are fully available without restriction. All relevant data are within the manuscript and its Supplementary Materials.

## Supplementary Materials

The following supporting information can be downloaded at: https://www.mdpi.com/article/10.3390/ani13193085/s1. PRISMA 2020 Checklist S1. Preferred Reporting Items for Systematic reviews and Meta-Analyses. (DOCX). Text S1. Formatted search strings–for Web of Science, CAB Direct, PubMed, Dissertations and theses. (DOCX). Text S2. Title/Abstract Level 1 relevance screening questions. Full text Level 2 relevance screening questions. (DOCX). Text S3. Calculation of standardized mean differences (Hedges’g) in nasal swab virus titers. (DOCX). Text S4: Steps 1 to 8–R code for meta-analyses. (DOCX). Text S5: Serum Hemagglutination Inhibition (HI) Titers (qualitative synthesis)–Indirect measure of protection: Additional explanation. (DOCX). Text S6. VEARD, IAV-S vaccine-induced MDI, and IAV-S vaccines in swine challenge trials: a narrative summary. (DOCX). Text S7. Outcome 5. Mean difference (MD) in average daily gain: Additional explanation (Direct measure of infection–non-specific endpoint). (DOCX). Text S8. Outcome 6. Coughing (insufficient data for meta-analysis): Additional explanation (Direct measure of infection–non-specific endpoint). (DOCX). Table S1. Raw data for meta-analyses–steps 01–08. (DOCX). Table S2. Study Design and Sampling Schedules (by sample type). (XLXS). Table S3. Antigenic characterization of maternal vaccination and challenge programs/study arm. (XLXS). Table S4. Antigenic characterization of concurrent piglet vaccination and challenge programs/study arm. (XLXS). Table S5. Overall Risk of Bias (ROB) for 15 eligible MDI challenge trials across five domains (ROB1-R OB5) (XLXS). Table S6. Risk of bias assessment by individual study, by ROB domain, and by Outcome (XLXS). Table S7. GRADE Partial† evidence profile: IAV-S vaccination of sows for the protection of offspring against IAV-S challenge. Figure S1. Sub-group meta-analysis forest plot of effects of vaccine-derived MDI on virus titers in IAV-S challenged piglets: sub-grouping by sow vaccine homology with challenge virus. Composite effect sizes adjusted using an estimate of the high (h) correlation between repeated measures–comparisons with vaccinated offspring were excluded from the analysis. (DOCX). Figure S2. Sub-group meta-analysis forest plot of effects of vaccine-derived MDI on virus titers in IAV-S challenged piglets: sub-grouping by sow vaccine homology with challenge virus. Composite effect sizes were adjusted using an estimate of the low (l) correlation between repeated measures–comparisons with concurrently vaccinated offspring were included in the analysis. (DOCX). Figure S3. Sub-group meta-analysis forest plot of effects of vaccine-derived MDI on virus titers in IAV-S challenged piglets: sub-grouping by sow vaccine homology with challenge virus. Composite effect sizes were adjusted using an estimate of the high (l) correlation between repeated measures–comparisons involving concurrently vaccinated offspring were omitted from this analysis. (DOCX). Figure S4. Bar graph, stacked by challenge trials, comparing group mean hemagglutination inhibition (HI) titers at each sample collection time-point for MDI-negative versus MDI-positive groups of unvaccinated piglets for each of 11 IAV-S vaccine-induced MDI challenge trials. (DOCX). Figure S5. Bar graph, stacked by challenge trials, comparing group mean hemagglutination inhibition (HI) titers at each sample collection time-point for MDI-negative versus MDI-positive and concurrently IAV-S vaccinated piglets for each of six IAV-S vaccine-induced MDI challenge trials. (DOCX). Figure S6. Forest plot of effects of IAV-S vaccine-induced MDI on the difference in mean HI titers at challenge between MDI-positive vs. MDI-negative piglets at time of first and second IAV-S challenge. (DOCX). Figure S7. Random effects meta-analysis forest plot of effects of vaccine-derived MDI on the mean difference ADG (kg) in IAV-S challenged piglets following 1st challenge.
