# Supplementary material for: Does Vaccine-Induced Maternally-Derived Immunity Protect Swine Offspring against Influenza a Viruses? A Systematic Review and Meta-Analysis of Challenge Trials from 1990 to May 2021
Source: Animals (Basel). 2023 Oct 3;13(19):3085. doi: 10.3390/ani13193085 (PMC10571953; doi:10.3390/ani13193085)
Supplement: Supplementary file 1 [file animals-13-03085-s001.zip › Supplemental files/S1 Fig.pdf]

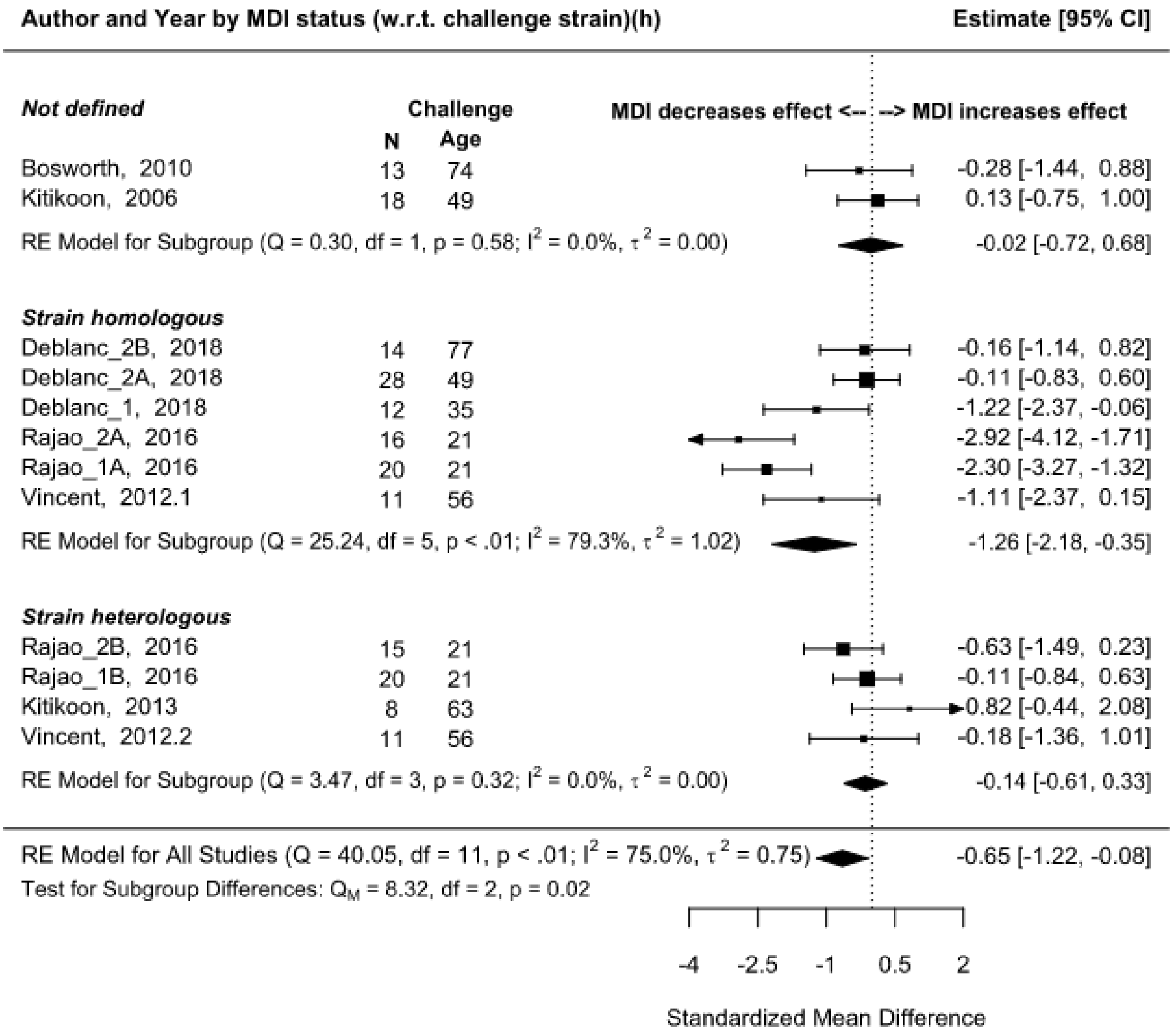

Figure S1. Sub-group meta-analysis forest plot of effects of vaccine-derived MDI on virus titres in IAV-S challenged piglets: sub-grouping by sow vaccine homology with challenge virus. Composite effect sizes adjusted using estimate of high (h) correlation between repeated measures – comparisons with vaccinated offspring were excluded from analysis.

MDI = maternally derived immunity in offspring as induced through IAV-S vaccination of dams. Meta-analysis sub-grouped by MDI status; Strain homologous = viral components of sow vaccine match the strain of the challenge virus; Strain heterologous = IAV-s virus antigenic components of the sow vaccine differ at the strain level from the challenge virus; Not defined = the IAV-S antigenic components of the maternal vaccine were not defined. N= total number of piglets in each treatment-control comparison; Challenge age is piglet days of age at challenge. Treatment-control comparisons involving concurrent IAV-S vaccination of piglet were not included in meta-analysis. Effect is mean virus titre (measured from nasal swab samples using virus isolation methods in all studies except for Bosworth et al. where virus quantified by PCR). Effect size is Hedges' g, (standardized mean difference corrected for small sample size bias) calculated as a composite of effect sizes derived by collapsing first across homologous treatment arms and then across repeated time points. An adjustment was made in calculations of pooled variances to account for assumed high (h) correlation (0.75) of measures from time point to time. Effect sizes are represented by squares with size proportional to their weighted contribution to the summary effect measure. values in the columns on the

right hand side equal the weighted contribution is shown as a %, effect sizes and the summary effect sizes under the header of Estimate with their corresponding 95% confidence intervals ([95% CI]Summary effects sizes are represented by diamonds. The dotted vertical line indicates a standardized mean difference of 0 (no effect difference between MDI positive and MDI negative groups). Points to the right of the line indicate MDI increases mean virus titres in offspring. I^2 95% uncertainty interval (lower bound, upper bound) = (49.20, 92.04).
