# Supplementary material for: Does Vaccine-Induced Maternally-Derived Immunity Protect Swine Offspring against Influenza a Viruses? A Systematic Review and Meta-Analysis of Challenge Trials from 1990 to May 2021
Source: Animals (Basel). 2023 Oct 3;13(19):3085. doi: 10.3390/ani13193085 (PMC10571953; doi:10.3390/ani13193085)
Supplement: Supplementary file 1 [file animals-13-03085-s001.zip › Supplemental files/S1 Text.pdf]

S1 Text. Formatted search strings*

For Web of Science:<br><br>((TS=(pork OR swine OR "Sus scrofa" OR pig OR pigs OR piglet OR piglets OR gilt OR gilts OR boar OR boars OR sow OR sows OR hog OR hogs OR “weaner pig” OR “weaned pig$” OR “feeder pig$” OR feeder OR feeders OR “finisher pig$” OR “finisher hog$” OR porcine OR “market-weight” NOT "guinea pig$") AND TS=(influenza OR IAV OR IAV$ OR flu OR SIV OR “H3N2” OR “H1N1” OR “H1N2” OR “H3N1” OR “H2N3”) AND TS=(immunize OR immuniz$ OR immunise OR immunis$ OR immunoprophylaxis OR intervention$ OR vaccinate OR vaccinat$ OR vaccine$ OR vaccine))) AND LANGUAGE: (English)<br>Indexes=SCI-EXPANDED, SSCI, A&HCI, CPCI-S, CPCI-SSH, ESCI<br>Timespan=2018-2021
For CAB Direct:<br><br>((((pork OR swine OR "Sus scrofa" OR pig OR pigs OR piglet OR piglets OR gilt OR gilts OR boar OR boars OR sow OR sows OR hog OR hogs OR “weaner pig” OR “weaned pig*” OR “feeder pig*” OR feeder OR feeders OR “finisher pig*” OR “finisher hog*” OR porcine NOT "guinea pig*") AND (influenza OR IAV OR IAV* OR flu OR SIV OR “H3N2” OR “H1N1” OR “H1N2” OR “H3N1” OR “H2N3”))) AND ((immunize OR immuniz* OR immunise OR immunis* OR immunoprophylaxis OR intervention* OR vaccinate OR vaccinat* OR vaccine OR vaccine*)) AND yr:[2018 TO 2021])
For PubMed:<br><br>(((pork[All Fields] OR ("swine"[MeSH Terms] OR "swine"[All Fields]) OR "Sus scrofa"[All Fields] OR ("swine"[MeSH Terms] OR "swine"[All Fields] OR "pig"[All Fields]) OR ("swine"[MeSH Terms] OR "swine"[All Fields] OR "pigs"[All Fields]) OR piglet[All Fields] OR piglets[All Fields] OR gilt[All Fields] OR gilts[All Fields] OR ("swine"[MeSH Terms] OR "swine"[All Fields] OR "boar"[All Fields]) OR ("swine"[MeSH Terms] OR "swine"[All Fields] OR "boars"[All Fields]) OR sow[All Fields] OR sows[All Fields] OR hog[All Fields] OR hogs[All Fields] OR "weaner pig"[All Fields] OR "weaned pigs"[All Fields] OR "feeder pig"[All Fields] OR feeder[All Fields] OR feeders[All Fields] OR "finisher pig"[All Fields] OR (finisher[All Fields] AND hog[All Fields]) OR ("swine"[MeSH Terms] OR "swine"[All Fields] OR "porcine"[All Fields]) NOT "guinea pig"[All Fields]) AND (("influenza, human"[MeSH Terms] OR ("influenza"[All Fields] AND "human"[All Fields]) OR "human influenza"[All Fields] OR "influenza"[All Fields]) OR IAV[All Fields] OR IAV_S[All Fields] OR ("influenza, human"[MeSH Terms] OR ("influenza"[All Fields] AND "human"[All Fields]) OR "human influenza"[All Fields] OR "flu"[All Fields]) OR SIV[All Fields] OR "H3N2"[All Fields] OR "H1N1"[All Fields] OR "H1N2"[All Fields] OR "H3N1"[All Fields] OR "H2N3"[All Fields])) AND (("immunisation"[All Fields] OR "vaccination"[MeSH Terms] OR "vaccination"[All Fields] OR "immunization"[All Fields] OR "immunization"[MeSH Terms]) OR immunized[All Fields] OR immunized[All Fields] OR ("immunization"[MeSH Terms] OR "immunization"[All Fields] OR "immunoprophylaxis"[All Fields]) OR ("methods"[MeSH Terms] OR "methods"[All Fields] OR "intervention"[All Fields]) OR interventions[All Fields] OR

vaccinated[All Fields] OR vaccinated[All Fields] OR ("vaccination"[MeSH Terms] OR "vaccination"[All Fields]) OR ("vaccination"[MeSH Terms] OR "vaccination"[All Fields] OR "vaccinations"[All Fields]) OR ("vaccines"[MeSH Terms] OR "vaccines"[All Fields] OR "vaccine"[All Fields]) OR ("vaccines"[MeSH Terms] OR "vaccines"[All Fields]))) AND ("2018/01/01"[CRDAT] : "3000"[CRDAT])
Dissertations and theses:<br><br>noft(pork OR swine OR "Sus scrofa" OR pig OR pigs OR piglet OR piglets OR gilt OR gilts OR boar OR boars OR sow OR sows OR hog OR hogs OR "weaner pig" OR "weaned pigs" OR "feeder pig" OR feeder OR feeders OR "finisher pig" OR "finisher hog" OR porcine OR "market-weight" NOT "guinea pig") AND noft(influenza OR IAV OR IAV-S OR flu OR SIV OR "H3N2" OR "H1N1" OR "H1N2" OR "H3N1" OR "H2N3") AND (immunize OR immunization OR immunise OR immunisation OR immunoprophylaxis OR vaccinate OR vaccination OR vaccines OR vaccine)Limits applied<br>Databases:32 databases searched<br>View list<br>These databases are searched for part of your query.<br>Limited by:Date: From 2018 January 01 to 2021<br>Source type:Dissertations & Theses<br>Document type:Dissertation/Thesis<br>Language:English

* Search strategy and search strings were developed and formatted for selected bibliometric platforms with support from University of Guelph librarians with systematic review methods expertise.
