# Supplementary material for: Does Vaccine-Induced Maternally-Derived Immunity Protect Swine Offspring against Influenza a Viruses? A Systematic Review and Meta-Analysis of Challenge Trials from 1990 to May 2021
Source: Animals (Basel). 2023 Oct 3;13(19):3085. doi: 10.3390/ani13193085 (PMC10571953; doi:10.3390/ani13193085)
Supplement: Supplementary file 1 [file animals-13-03085-s001.zip › Supplemental files/S2 Text.pdf]

S2 Text

Title/Abstract Level 1 relevance screening questions:

(1= Advance†, 0 = Exclude)

†Advance to next question, or if the last question, to the next level of screening.

1. Is this report/study/document about Influenza A virus in/from swine (IAV-S) where swine or direct applicability to swine is the focus (i.e. excludes studies of IAV-S in humans with variant IAV-S, or IAV-S in other species)?

   Yes 1, No 0, Unclear 1
2. Is the citation primary research?

   Yes 1
   No, it is an editorial or commentary. 0
   No, it is a white paper, working report, policy paper, issue paper, or guidelines 0
   *No, it is a review.
   No, it is another type of publication. 0
   Unclear 1

   ***3a)** What is the review type as indicated by the authors in the title/abstract?

   A traditional or narrative review. 0
   A systematic review without a meta-analysis 0
   A meta-analysis. 0
   A systematic review and meta-analysis. 0

* This is a conditional question applied only to citations identified as reviews.

3. Does this study involve vaccine research in swine where the unit of study is higher than the sub-animal level (e.g. not at the tissue, cellular, molecular, etc. level)?

   Yes 1, No 0, Unclear 1

4. Are sows (or first parity gilts) the study population vaccinated?

   Yes 1, No 0, Unclear 1
5. Is the full text available in English?

   Yes 1, No 0, Unclear 1

Full text Level 2 relevance screening questions:

(1= Advance†, 0 = Exclude)

† Advance to next question, or if the last question, to the next level of screening.

1. Is this report/study/document about Influenza A virus in/from swine (IAV-S) where swine or direct applicability to swine is the focus (i.e. excludes studies of IAV-S in humans with variant IAV-S, or IAV-S in other species)?

   Yes 1, No 0

2. Is the citation primary research?

   Yes 1
   No, it is an editorial or commentary. 0

No, it is a white paper, working report, policy paper, issue paper, or guidelines 0
No, it is a review*. 1
No, it is another type of publication. 0

***3a)** What is the review type as indicated by the authors in the title/abstract?
(This is a conditional question and applied only to citations identified as reviews.)
A traditional or narrative review. 0
A systematic review without a meta-analysis 0
A meta-analysis. 0
A systematic review and meta-analysis. 0

3. Does this study involve vaccine research in swine where the unit of study is higher than the sub-animal level (e.g. not at the tissue, cellular, molecular, etc. level)?
   Yes 1, No 0

4. Are sows (or first parity gilts) the study population vaccinated?
   Yes 1, No 0

5. Are outcomes measured in offspring of vaccinated dams?
   Yes 1, No 0

6. Is there an offspring comparison group?
   Yes 1, No 0

7. Is the study a challenge trial?
   Yes 1 No 1

8. Was at least one of the following offspring outcomes reported?
   - serum hemagglutination inhibition (HAI) titres
   - virus detection, duration of virus, or virus titres sampled from oropharyngeal or nasal swab
   - Average daily gain (ADG)
   - Coughing (measured at the group level)

   Yes 1 No 1
