# Supplementary material for: Does Vaccine-Induced Maternally-Derived Immunity Protect Swine Offspring against Influenza a Viruses? A Systematic Review and Meta-Analysis of Challenge Trials from 1990 to May 2021
Source: Animals (Basel). 2023 Oct 3;13(19):3085. doi: 10.3390/ani13193085 (PMC10571953; doi:10.3390/ani13193085)
Supplement: Supplementary file 1 [file animals-13-03085-s001.zip › Supplemental files/S3 Fig.pdf]

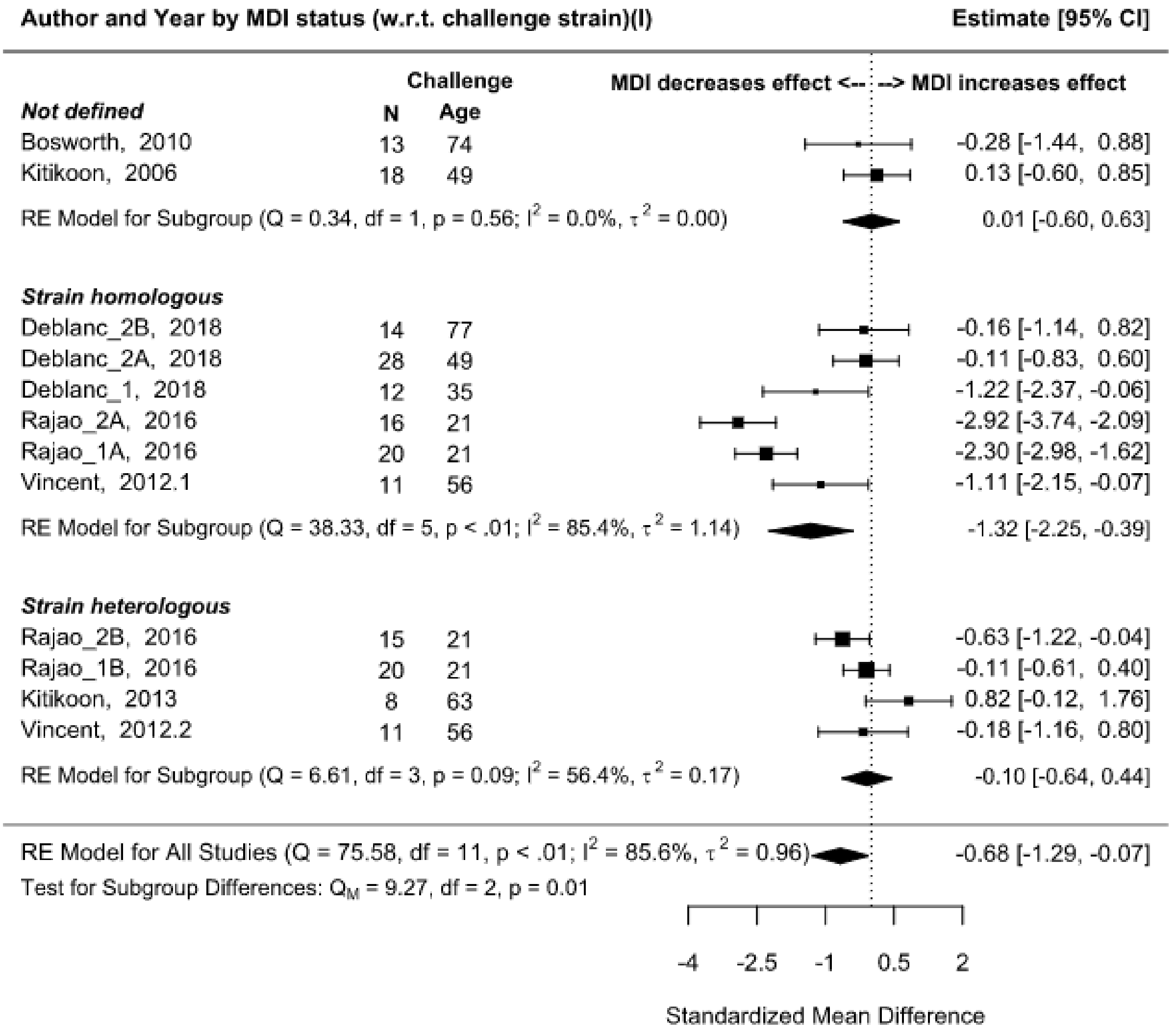

Figure S3. Sub-group meta-analysis forest plot of effects of vaccine-derived MDI on virus titres in IAV-S challenged piglets: sub-grouping by sow vaccine homology with challenge virus. Composite effect sizes adjusted using estimate of high (l) correlation between repeated measures – comparisons involving concurrently vaccinated offspring were omitted from this analysis.

MDI = maternally derived immunity in offspring as derived through vaccination of dams against IAV-S; N= total number of piglets in each treatment-control comparison; Challenge age is days of age at challenge. Meta-analysis sub-grouped by MDI status; Strain homologous = viral components of sow vaccine match the strain of the challenge virus; Strain heterologous = vaccine components of the sow vaccine are from an IAV-s virus that differs at the strain level from the challenge virus; Not defined = the IAV-S antigenic components of the maternal vaccine were not defined. Treatment-control comparisons involving concurrent IAV-S vaccination of piglet were not included in meta-analysis. Treatment-control comparisons involving concurrent IAV-S vaccination of piglet were not included in meta-analysis. Effect is mean virus titre (measured from nasal swab samples using virus isolation methods in all studies except for Bosworth et al. where virus was quantified by PCR). Effect size is Hedges' g, (standardized mean difference corrected for small sample size bias) calculated as a composite of effect sizes derived by collapsing first across homologous treatment arms and then across repeated time points. An adjustment was made in calculations of pooled variances to account for assumed high (l) correlation (0.2) of measures from time point to time point. Columns on the right under the heading of Estimate are the values and their 95% confidence intervals for the corresponding effect sizes and summary effect sizes. Effect sizes are represented by squares with size proportional to their weighted contribution to the summary effect measure. Diamonds equal summary effects sizes for each of the

sub-groups and the overall effect. The dotted vertical line indicates a standardized mean difference of 0 (no effect difference between MDI positive and MDI negative groups). Points to the right of the line indicate MDI increases the effect in the treatment group. I^2 95% uncertainty interval (lower bound, upper bound) = (70.51, 94.96).
