# Supplementary material for: Does Vaccine-Induced Maternally-Derived Immunity Protect Swine Offspring against Influenza a Viruses? A Systematic Review and Meta-Analysis of Challenge Trials from 1990 to May 2021
Source: Animals (Basel). 2023 Oct 3;13(19):3085. doi: 10.3390/ani13193085 (PMC10571953; doi:10.3390/ani13193085)
Supplement: Supplementary file 1 [file animals-13-03085-s001.zip › Supplemental files/S3 Text.pdf]

S3 Text
Calculation of standardized mean differences (Hedges'g) in nasal swab virus titres.

n1 and n2 are group sizes
S1 and S2 are group standard deviations
V is variance
SE is standard error
d = sample estimate of the standardized mean difference between 2 independent groups (also called Cohen's d)
J = correction factor to convert d to Hedges' g to adjust for small sample bias
df = degrees of freedom n1 + n2 - 2

All formulas from Chapter 4(Borenstein *et. al*., 2009): Effect Sizes Based on Means, pages 22-27.

Calculation of Hedges g from reported group size, mean and measures of variance as provided.
Standard mean differences (d) calculated as follows:

$$d = \frac{\overline{X}_1 - \overline{X}_2}{S_{within}}.$$

The within group standard deviation pooled between groups is calculated as

$$S_{within} = \sqrt{\frac{(n_1 - 1)S_1^2 + (n_2 - 1)S_2^2}{n_1 + n_2 - 2}}$$

The variance of d was calculated using

$$V_d = \frac{n_1 + n_2}{n_1 n_2} + \frac{d^2}{2(n_1 + n_2)}.$$

And the standard error of d is

$$SE_d = \sqrt{V_d}.$$

And

$$J = 1 - \frac{3}{4df - 1}.$$

Then

$$g = J \times d,$$

$$V_g = J^2 \times V_d,$$

And

$$SE_g = \sqrt{V_g}.$$

Formulas for computing the composite effect sizes and variances

Formulas for combining effect sizes (Hedges g) across treatment groups and across multiple time-points

From Borenstein et al 2011, Chapter 24: Multiple Outcomes or Time-Points within a Study, pg.225-238.

Y1 effect size from outcome 1
Y2 effect size from outcome 2
Yi = is the effect size for variable I =1, …m
m = the number of outcomes in a study
r = correlation coefficient for Y1 and Y2 where r ranges from 0 to 1 and r=0 if Y1 and Y2 are unrelated
If r=0.5 the variance is ~ the average to the two variances.
Vi = variance of Yi
$\overline{Y}$ = composite effect size

The correlation amongst outcomes at different time points was unknown but assumed to be 0.5 between different MDA positive treatment arms and a sensitivity analysis was performed calculating variances using both a low (r=0.2) and a high (r=0.75) estimate of correlation.

Effect sizes were combined for studies involving multiple MDA positive treatment arms and differing but concurrent piglet vaccination. Effect sizes were also combined for mean differences in virus titres as measured from repeated collection of nasal swabs over the study period (i.e. multiple time-points).

Formulas for computing the composite effect size (pg230):

$$\overline{Y} = \frac{1}{m}\left(\sum_{j}^{m} Y_j\right)$$

Variances of the mean of two correlated variables calculated using (pg. 228)

$$\text{var}\left(\frac{1}{2}(Y_1 + Y_2)\right) = \left(\frac{1}{2}\right)^2 \text{var}(Y_1 + Y_2) = \frac{1}{4}\left(V_1 + V_2 + 2r\sqrt{V_1}\sqrt{V_2}\right)$$

Variances of the mean of several correlated variables were calculated using (pg. 228)

$$\text{var}\left(\frac{1}{m}\sum_{i=1}^{m} Y_i\right) = \left(\frac{1}{m}\right)^2 \text{var}\left(\sum_{i=1}^{m} Y_i\right) = \left(\frac{1}{m}\right)^2 \left(\sum_{i=1}^{m} V_i + \sum_{i \neq j}\left(r_{ij}\sqrt{V_i}\sqrt{V_j}\right)\right)$$

Borenstein, M., Hedges, L. V., Higgins, J.P.T., Rothstein, H.R., 2009. Introduction to Meta-Analysis. John Wiley and Sons, Ltd, Padstow, Cornwall.
