# Supplementary material for: Does Vaccine-Induced Maternally-Derived Immunity Protect Swine Offspring against Influenza a Viruses? A Systematic Review and Meta-Analysis of Challenge Trials from 1990 to May 2021
Source: Animals (Basel). 2023 Oct 3;13(19):3085. doi: 10.3390/ani13193085 (PMC10571953; doi:10.3390/ani13193085)
Supplement: Supplementary file 1 [file animals-13-03085-s001.zip › Supplemental files/S4 Fig.pdf]

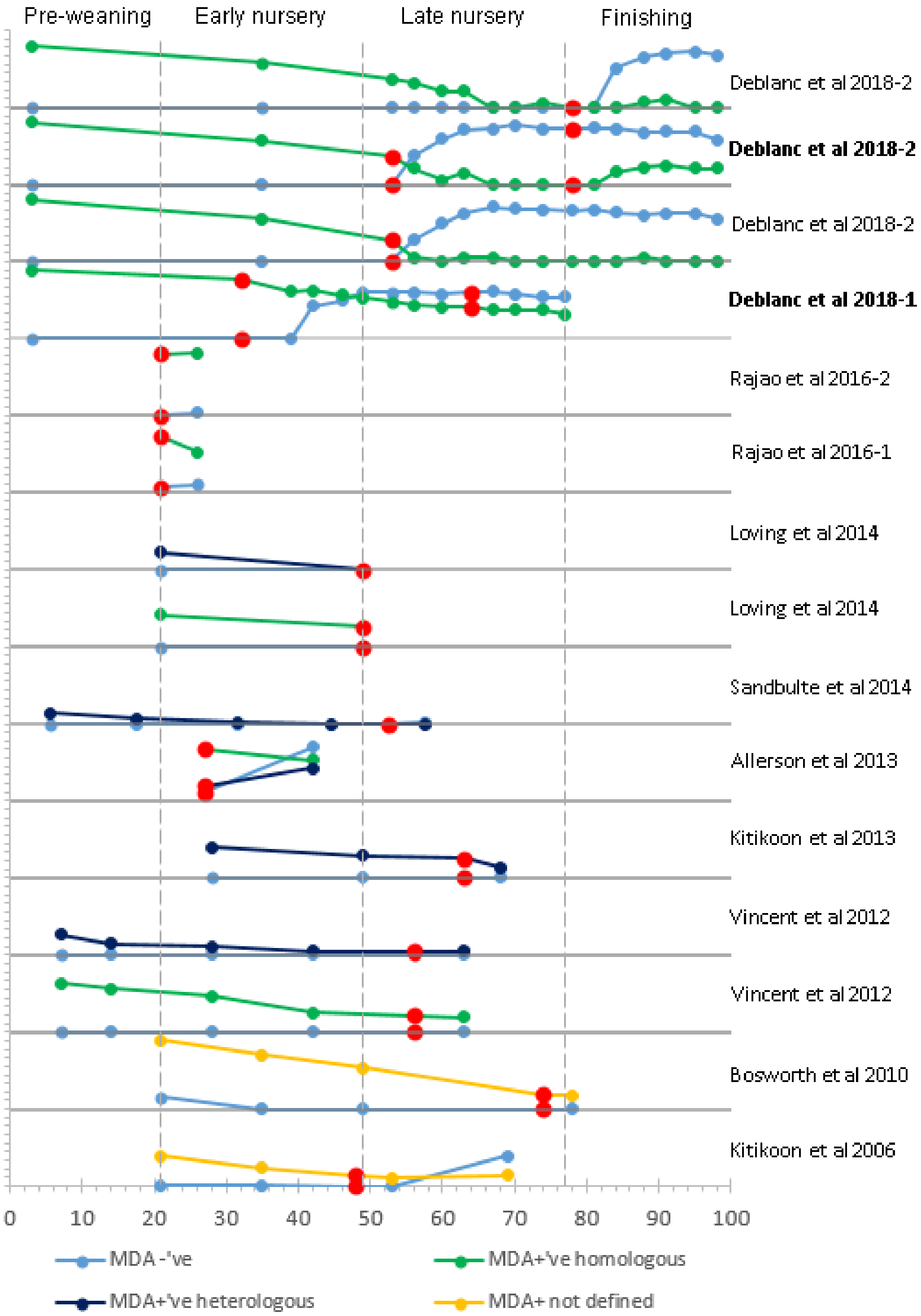

Figure S4. Bar graph, stacked by challenge trials, comparing group mean hemagglutination inhibition (HI) titres at each sample collection time-point for MDI negative versus MDI positive groups of unvaccinated piglets for each of 11 IAV-S vaccine-induced MDI challenge trials.

MDI = vaccine-induced maternally-derived immunity and in this figure is labelled as MDA (maternally derived antibodies); HI assay antiserum is against challenge virus; study arms with concurrent piglet vaccination are not shown (see ***S***Fig 12); studies ordered by publication date; MDI homology = antigenic match of the maternal vaccine composition to the challenge virus strain (heterologous or homologous); MDA-'ve = MDI negative control group piglets; MDA+'ve heterologous = MDI positive piglets from sows vaccined with a vaccine heterologous to the challenge virus; MDA+'ve homologous = MDI positive piglets from sows vaccined with a vaccine homologous to the challenge virus; MDA+ not defined = MDI positive piglets from vaccinated sows with no information provided on vaccine antigens; X axis = piglet days of age, Y-axis (unlabelled) = Log2 reciprocal geometric mean titres (GMT) with stacked studies each on the same scale of 0 to 9 (equates to HAI titres <1:10 to 1:2560); grey vertical dashed lines equal 3, 7 and 11 weeks of age; large red circle markers indicate timing of piglet challenge; author and publication years in **bold** for studies involving a second IAV-S challenge of piglets.
