# Supplementary material for: Does Vaccine-Induced Maternally-Derived Immunity Protect Swine Offspring against Influenza a Viruses? A Systematic Review and Meta-Analysis of Challenge Trials from 1990 to May 2021
Source: Animals (Basel). 2023 Oct 3;13(19):3085. doi: 10.3390/ani13193085 (PMC10571953; doi:10.3390/ani13193085)
Supplement: Supplementary file 1 [file animals-13-03085-s001.zip › Supplemental files/S4 Text.pdf]

S4 Text Steps 1_8 R code for meta-analyses
Summary of steps (R code for each step is detailed below)

Step 01 – Virus titres from nasal swabs
Meta-analysis using R metaphor package (rma function)
Effect size = SMD (Standardized mean difference)
Input data = Hedge's g and variance pre-calculated from reported arm level data (as continuous data) (See Supplemental File 4)

Step 02 –Analysis of virus titre data for small study publication bias
- funnel plots, trim and fill
Input data as per Step 01

Step 03 –Virus detection = virus isolation or PCR methods from nasal swabs
Survival analysis using R survival and survminer packages
Input data = reported raw individual piglet days to event (start / stop shedding)
Cox proportional Hazard ratios and standard errors computed for each study generating 'yi' and 'sei' variables for use in Step 04.

Step 04 – Virus detection in nasal swabs
Meta-analysis using R metaphor package (rma function)
Effect size = HR (Hazard Ratios)
Input data = HR and Standard error (SE) calculated in Step 03

Step 05 - Virus detection in nasal swabs (time to start and time to stop shedding)
Meta-analysis using R metaphor package (escalc & rma functions)
Effect Size = MD (Mean Difference)
Input Data = -group size, mean, and standard deviation (as continuous data), pre-calculated from individual pig data

Step06 - Virus detection in nasal swabs
Meta-analysis using R metaphor package (metabin function)
Effect size = RR (Risk Ratio)
Input data = extracted as treatment group 2x2 table (binary data)

Step07 – Average daily gain (ADG)
Meta-analysis using R metaphor package (escalc & rma functions)
Effect Size = MD (Mean Difference)
Input Data = group size, mean, and standard deviation (as continuous data)

Step08 – Hemagglutinin Inhibition titres (HAI)
Meta-analysis using R metaphor package (escalc functions only)
Effect = MD (Mean Difference in reciprocal log2 HAI change from baseline where baseline = earliest sampling period post- challenge – no prior contemporary sampling)
Input Data = group size, mean HAI scores, and standard deviation (as continuous data)

Step 01 – Virus titres from nasal swabs
Meta-analysis using R metaphor package (rma function)

Effect size = SMD (Standardized mean difference)
Input data = Hedge's g and variance pre-calculated from reported arm level data (as continuous data)

Standardized group mean differences were calculated using a bias correction for small samples sizes (Hedges'g). Effect sizes were combined across treatment groups and time points if studies reported multiple treatment arms and/ or sampling time points using formulas as outlined in Chapters 4 and 24 of Borenstein et al (2009) (see appendix X for additional details). Variances for composite time point measures were calculated using as estimate of high (r=0.75) and of low (r=0.2) correlation. Meta-analyses were conducted using each of the calculated variances.

```
setwd("C:/Users/sheila/Documents/PhD/Chapters/Chapter_3_data")

## load required package
library(metafor)

library(meta)
library(graphics)
library(ggplot2)
##### Individual animal virus tire measures were collected at multiple time points###
##### and were combined where and variances derived assuming either high correlation###
##### or low correlation between time points measures, therefore 2 data sets are used - ###
##### high (estimated correlation =.75) and low (estimated corr. = .2)###

#####  analysis of moderator of MDA strain homology with challenge virus- ALL studies (i.e. VX and NV)
####
##### analyze high correlation data ####

vtdatah<-read.csv("vt_MA_data_h.csv", header=T)

head(vtdatah)
dim(vtdatah)
str(vtdatah)

### fit random-effects model of MA of all trials-using high correlation data-h
vtmetadatah <- rma(yi,vi,data=vtdatah, slab=paste(Author, Year, sep=",  "))
vtmetadatah$I2
confint(vtmetadatah)
summary(vtmetadatah)
influence(vtmetadatah)

### a helper function to add Q-test, I^2, and tau^2 estimate info
mlabfun <- function(text, vtmetadatah) {
  list(bquote(paste(.(text),
             " (Q = ", .(formatC(vtmetadatah$QE, digits=2, format="f")),
             ", df = ", .(vtmetadatah$k - vtmetadatah$p),
             ", p ", .(metafor:::.pval(vtmetadatah$QEp, digits=2, showeq=TRUE, sep=" ")), "; ",
             I^2, " = ", .(formatC(vtmetadatah$I2, digits=1, format="f")), "%, ",
             tau^2, " = ", .(formatC(vtmetadatah$tau2, digits=2, format="f")), ")")))}

### Turn on graphics device and adjust dimensions
svg(file='forestvtMDAh.svg')
```

```
### set up the forests plot (with extra rows of information about)
forest(vtmetadatah,ilab=cbind(vtdatah$tot_n, vtdatah$Chal_age, vtdatah$PgVx_strain),
      ilab.xpos=c(-10.5,-9.0,-7.0), xlim=c(-19, 6.5),cex=0.8, ylim=c(-1.5, 32),alim = c(-4,2), order =
vtdatah$MDA_strain,rows=c(3:8,13:19,24:27),
      mlab=mlabfun("RE Model for All Studies", vtmetadatah), xlab = "Standardized Mean Difference",
      header="Author and Year by MDI status (w.r.t. challenge strain)(h)")

### set font expansion factor (as in forest() above) and use a bold font
op <- par(cex=0.75, font=2)

### add additional column headings to the plot
text(c(-10.5,-9.0,-7.0), 28, c("N", "Age", " Vx"))
text(c(-9.0,-7.0), 29, c("Challenge","Piglet"))
text(c(0),29,c(" MDI increases effect <--   --> MDI decreases effect "))

### switch to bold italic font
par(font=4)

### add text for the subgroups
text(-19, c(28,20,9), pos=4, c("Not defined",
                          "Strain homologous",
                          "Strain heterologous"))

### set par back to the original settings
par(op)

### fit random-effects model in the three subgroups
vtmetadatah.n <- rma(yi, vi, subset=(MDA_strain=="nd"), data=vtdatah)
vtmetadatah.m <- rma(yi, vi, subset=(MDA_strain=="hom"), data=vtdatah)
vtmetadatah.t <- rma(yi, vi, subset=(MDA_strain=="het"),  data=vtdatah)

### add summary polygons for the three subgroups
addpoly(vtmetadatah.n, row=22.5, cex=0.75, mlab=mlabfun("RE Model for Subgroup", vtmetadatah.n))
addpoly(vtmetadatah.m, row= 11.5, cex=0.75, mlab=mlabfun("RE Model for Subgroup", vtmetadatah.m))
addpoly(vtmetadatah.t, row= 1.5, cex=0.75, mlab=mlabfun("RE Model for Subgroup", vtmetadatah.t))

### fit meta-regression model to test for subgroup differences
vtmetadatah <- rma(yi, vi, mods = ~ MDA_strain, data=vtdatah)

### add text for the test of subgroup differences
text(-19, -2.2, pos=4, cex=0.75, bquote(paste("Test for Subgroup Differences: ",
                                   Q[M], " = ", .(formatC(vtmetadatah$QM, digits=2, format="f")), ", df = ",
.(vtmetadatah$p - 1),
                                   ", p = ", .(formatC(vtmetadatah$QMp, digits=2, format="f")))))
### Turn off graphics device - plot will be saved automatically to file but not shown on plot window
dev.off()


####Creating a funnel plot and trim and fill here ########
funnel(vtmetadatah, main = "Standard Error (virus titre-h)")

#TRIM and FILL analysis
trimfill(vtmetadatah)

####################################################
```

```
##### analysis of moderator of MDA strain homology with challenge virus- NO vaccine studies (i.e.NV only) ####

##### analyze high correlation data ####

vtdatah<-read.csv("vt_MA_data_h.csv", header=T)

head(vtdatah)
dim(vtdatah)
str(vtdatah)

### fit random-effects model of MA of NV trials - h##

vtNVh<- vtdatah[vtdatah$piglet=="NV",]

vtmetaNVh <- rma(yi,vi,data=vtNVh, slab=paste(Author, Year, sep=",  "))
vtmetaNVh$I2
confint(vtmetaNVh)
summary(vtmetaNVh)
influence(vtmetaNVh)

### a helper function to add Q-test, I^2, and tau^2 estimate info
mlabfunNV <- function(text, vtmetaNVh) {
  list(bquote(paste(.(text),
                  " (Q = ", .(formatC(vtmetaNVh$QE, digits=2, format="f")),
                  ", df = ", .(vtmetaNVh$k - vtmetaNVh$p),
                  ", p ", .(metafor:::.pval(vtmetaNVh$QEp, digits=2, showeq=TRUE, sep=" ")), "; ",
                  I^2, " = ", .(formatC(vtmetaNVh$I2, digits=1, format="f")), "%, ",
                  tau^2, " = ", .(formatC(vtmetaNVh$tau2, digits=2, format="f")), ")")))}

### Turn on graphics device and adjust dimensions
svg(file='forestvtMD_NVh.svg')

### set up the forests plot (with extra rows of information about)
forest(vtmetaNVh,ilab=cbind(vtNVh$tot_n, vtNVh$Chal_age),
      ilab.xpos=c(-10.5,-9.0), xlim=c(-19, 6.5),cex=0.8, ylim=c(-1.5, 28),alim = c(-4,2), order =
vtNVh$MDA_strain,rows=c(3:6,11:16,21:22),
      mlab=mlabfunNV("RE Model for All Studies", vtmetaNVh), xlab = "Standardized Mean Difference",
      header="Author and Year by MDI status (w.r.t. challenge strain)(h)")

### set font expansion factor (as in forest() above) and use a bold font
op <- par(cex=0.75, font=2)

### add additional column headings to the plot
text(c(-10.5,-9.0), 23, c("N", "Age"))
text(c(-9.0), 24, c("Challenge"))
text(c(0),24,c("    MDI increases effect <--    --> MDI decreases effect "))

### switch to bold italic font
par(font=4)

### add text for the subgroups
text(-19, c(24,17,7), pos=4, c("Not defined",
                         "Strain homologous",
                         "Strain heterologous"))
```

```
### set par back to the original settings
par(op)

### fit random-effects model in the three subgroups
vtmetaNVh.n <- rma(yi, vi, subset=(MDA_strain=="nd"), data=vtNVh)
vtmetaNVh.m <- rma(yi, vi, subset=(MDA_strain=="hom"), data=vtNVh)
vtmetaNVh.t <- rma(yi, vi, subset=(MDA_strain=="het"),  data=vtNVh)

### add summary polygons for the three subgroups
addpoly(vtmetaNVh.n, row=19.5, cex=0.75, mlab=mlabfunNV("RE Model for Subgroup", vtmetaNVh.n))
addpoly(vtmetaNVh.m, row= 9.5, cex=0.75, mlab=mlabfunNV("RE Model for Subgroup", vtmetaNVh.m))
addpoly(vtmetaNVh.t, row= 1.5, cex=0.75, mlab=mlabfunNV("RE Model for Subgroup", vtmetaNVh.t))

### fit meta-regression model to test for subgroup differences
vtmetaNVh <- rma(yi, vi, mods = ~ MDA_strain, data=vtdatah)

### add text for the test of subgroup differences
text(-19, -2.2, pos=4, cex=0.75, bquote(paste("Test for Subgroup Differences: ",
                                     Q[M], " = ", .(formatC(vtmetaNVh$QM, digits=2, format="f")), ", df = ",
.(vtmetaNVh$p - 1),
                                     ", p = ", .(formatC(vtmetaNVh$QMp, digits=2, format="f")))))
### Turn off graphics device - plot will be saved automatically to file but not shown on plot window
dev.off()

funnel(vtmetaNVh, main = "Standard Error (virus titre-nvh)")

##### continue analyzing using low correlation data set ####
```

Step 02 –Analysis of virus titre data for small study publication bias - funnel plots, trim and fill

Input data as per Stop 01

```
#####analysis for small study publication bias - funnel plots ######

setwd("C:/Users/sheila/Documents/PhD/Chapters/Chapter_3_data")

## load required package
library(metafor)
library(meta)
library(graphics)

##### Individual animal virus tire measures were collected at multiple time points###
##### and were combined where and variances derived assuming either high correlation###
##### or low correlation between time points measures, therefore 2 data sets are used - ###
##### high (estimated correlation =.75) and low (estimated corr. = .2)###

#####  analysis of - ALL studies (i.e. VX and NV) ####
##### analyze high correlation data ####

vtdatah<-read.csv("vt_MA_data_h.csv", header=T)

vtmetadatah <- rma(yi,vi,data=vtdatah, slab=paste(Author, Year, sep=",  "))

forest(vtmetadatah, order = "obs",showweights = TRUE, header = TRUE,)
```

```
funnel(vtmetadatah, main = "Standard Error (virus titre-h)")

trimfill(vtmetadatah)
vtmetadatahi<-influence(vtmetadatah)
plot(vtmetadatahi)
head(vtmetadatahi)
dim(vtmetadatahi)
str(vtmetadatahi)
qqnorm(vtmetadatah)
baujat(vtmetadatah)
leave1out(vtmetadatah)
#####  analysis of - studies without piglet vaccination (i.e. only NV) ####
##### continue analyzing using low correlation data set ####
```

Step 03 –Virus detection = virus isolation or PCR methods from nasal swabs
Survival analysis using R survival and survminer packages
Input data = reported raw individual piglet days to event (start / stop shedding)

- Hazard ratios and standard errors computed for each study generating ‘yi’ and ‘sei’ variables for use in Step 04.

```
### source  of code: http://www.sthda.com/english/wiki/survival-analysis-basics

setwd("C:/Users/sheila/Documents/PhD/Chapters/Chapter_3_data")

## for cox's regression
install.packages(c("survival", "survminer"))

library("survival")
library("survminer")
library(graphics)
library(ggplot2)

### determine Kaplan-Meier survival curves for each study to be included in meta-analysis

study.3622hm.df<-read.csv("3622hom_dur_of_shed_raw_data.csv", header=T)

head(study.3622hm.df)
fit.3622hm.df <- survfit(Surv(time_to_shedding, start_shedding) ~ Treatment, data = study.3622hm.df)
print(fit.3622hm.df)

# Summary of survival curves
summary(fit.3622hm.df)
# Access to the sort summary table
summary(fit.3622hm.df)$table

d.3622hm <- data.frame(time = fit.3622hm.df$time,
                  n.risk = fit.3622hm.df$n.risk,
                  n.event = fit.3622hm.df$n.event,
                  n.censor = fit.3622hm.df$n.censor,
                  surv = fit.3622hm.df$surv,
```

```
                    upper = fit.3622hm.df$upper,
                    lower = fit.3622hm.df$lower
)
head(d.3622hm)

## survival curves

# Change color, linetype by strata, risk.table color by strata
ggsurvplot(fit.3622hm.df,
          pval = TRUE, conf.int = TRUE,mark.time=TRUE,
          risk.table = TRUE, # Add risk table
          risk.table.col = "strata", # Change risk table color by groups
          linetype = "strata", # Change line type by groups
          surv.median.line = "hv", # Specify median survival
          ggtheme = theme_bw(), # Change ggplot2 theme
          palette = c("green", "red", "cyan"))

### Cox's model
### simple univariable analysis
cox.3622hm <- coxph(Surv(time_to_shedding, start_shedding) ~ Treatment, data = study.3622hm.df)
cox.3622hm

summary(cox.3622hm)

##################################################
# repeat for 3622hom for time to stop shedding
##################################################

study.3622shm.df<-
read.csv("C:/Users/sheila/Documents/PhD/Chapters/Chapter_3_data/3622hom_dur_of_shed_raw_data.c
sv", header=T)

head(study.3622shm.df)

fit.3622shm.df <- survfit(Surv(time_to_stop_shedding, stop_shedding) ~ Treatment, data =
study.3622shm.df)
### Cox's model

### simple univariable analysis
cox.3622shm <- coxph(Surv(time_to_stop_shedding, stop_shedding) ~ Treatment, data =
study.3622shm.df)
cox.3622shm

summary(cox.3622shm)

########################################################
####Repeat time to shedding, and time to stop shedding analyses for each study treatment arm
########################################################
```

Step 04 – Virus detection in nasal swabs
Meta-analysis using R metaphor package (rma function)
Effect size = HR (Hazard Ratios)
Input data = HR and Standard error (SE) calculated in Step 03

```
setwd("C:/Users/sheila/Documents/PhD/Chapters/Chapter_3_data")

## load required package
library(metafor)
library(meta)
library(graphics)
library(ggplot2)

#####  analysis of time to shedding using hazard ratios derived in step03 ####
#### time_to_shed_MA_data.csv compiled by extracting manually from results of step03 survival
analyses

t2shed<-read.csv("time_to_shed_MA_dataV2.csv", header=T)

head(t2shed)
dim(t2shed)
str(t2shed)

### fit random-effects model of MA of all trials

t2shedmeta <- rma(yi,sei=sei,data=t2shed, slab=paste(Author, Year, sep=",  "))
t2shedmeta$I2
confint(t2shedmeta)
summary(t2shedmeta)
influence(t2shedmeta)
summary(t2shedmeta$vi)
plot(density(t2shedmeta$vi))

### a helper function to add Q-test, I^2, and tau^2 estimate info
mlabfun <- function(text, t2shedmeta) {
  list(bquote(paste(.(text),
                " (Q = ", .(formatC(t2shedmeta$QE, digits=2, format="f")),
                ", df = ", .(t2shedmeta$k - t2shedmeta$p),
                ", p ", .(metafor:::.pval(t2shedmeta$QEp, digits=2, showeq=TRUE, sep=" ")), "; ",
                I^2, " = ", .(formatC(t2shedmeta$I2, digits=1, format="f")), "%, ",
                tau^2, " = ", .(formatC(t2shedmeta$tau2, digits=2, format="f")), ")")))}

### set up the forests plot (with extra rows of information about)
forest(t2shedmeta,showweights=TRUE,ilab=cbind(t2shed$tot_n, t2shed$Chal_age, t2shed$MDA_strain),
      ilab.xpos=c(-9.5,-8.0,-6.5), xlim=c(-8, 11),cex=1, ylim=c(-1.0, 13),alim = c(-2,2), order =
t2shed$MDA_strain,rows=c(1,3:7),
      mlab=mlabfun("RE Model for All Studies", t2shedmeta), xlab = "Log Hazard Ratio",
      header="Author and Year - Virus Shedding")

### set font expansion factor (as in forest() above) and use a bold font
op <- par(cex=0.9, font=2)

### add additional column headings to the plot
text(c(-9.5,-8.0,-6.5), 8.0, c("N", "Age", " MDI"))
text(c(-8.0), 9, c("Challenge"))
text(c(0),10,c("    MDI increases effect <--    --> MDI decreases effect "))

### fit random-effects model in the three subgroups
t2shedmeta.m <- rma(yi, sei=sei, subset=(MDA_strain=="hom"), data=t2shed)

### add summary polygons for the three subgroups
```

```
addpoly(t2shedmeta.m, row= 2, cex=0.8, mlab=mlabfun("RE Model for Subgroup", t2shedmeta.m))

### fit meta-regression model to test for subgroup differences
t2shedmeta <- rma(yi, sei=sei, mods = ~ MDA_strain, data=t2shed)

#####################################################
#####  repeat as above for time_to_stop_shedding using hazard ratios derived in step03 ####
#####################################################

t2stop<-read.csv("time_to_stop_shed_MA_data.csv", header=T)
```

Step 05 - Virus detection in nasal swabs
Meta-analysis using R metaphor package (escalc & rma functions)
Effect Size = SMD (Standard Mean Difference - as Hedges g)

- (The metacont function from R package meta uses Hedges's g based on the pooled sample variance -see explained in Schwarzer, Carpenter & Rucker (Meta-Analysis with R, 2015, pp. 25)

Input Data = -group size, mean, and standard deviation (as continuous data)

- pre-calculated from individual pig data

```
setwd("C:/Users/sheila/Documents/PhD/Chapters/Chapter_3_data")

## load required package
library(metafor)
library(meta)
library(graphics)
library(ggplot2)

#####  analysis of time to shedding using standardized mean differences

t2shedmd<-read.csv("t2shed_smd_manual.csv", header=T)

head(t2shedmd)
dim(t2shedmd)
str(t2shedmd)
dat1shed <- escalc(measure="SMD", m1i=m1i, sd1i=sd1i, n1i=n1i,
            m2i=m2i, sd2i=sd2i, n2i=n2i, data=t2shedmd)
dat1shed

res1 <- rma(yi,vi,data=dat1shed, slab=paste(Author, Year, sep=",  "))
res1
res1$I2
confint(res1)
summary (res1)
influence(res1)

### a helper function to add Q-test, I^2, and tau^2 estimate info
mlabfun <- function(text, res1) {
  list(bquote(paste(.(text),
                " (Q = ", .(formatC(res1$QE, digits=2, format="f")),
                ", df = ", .(res1$k - res1$p),
```

```
        ", p ", .(metafor:::.pval(res1$QEp, digits=2, showeq=TRUE, sep=" ")), "; ",
        I^2, " = ", .(formatC(res1$I2, digits=1, format="f")), "%, ",
        tau^2, " = ", .(formatC(res1$tau2, digits=2, format="f")), ")")))}

### set up the forests plot (with extra rows of information about)
forest(res1,showweights=TRUE,ilab=cbind(dat1shed$tot_n, dat1shed$Chal_age, dat1shed$MDA_strain),
    ilab.xpos=c(-6.5,-5.0,-3.5), xlim=c(-11, 9.5),cex=.9, ylim=c(-1.5, 12),alim = c(-2,6), order =
dat1shed$MDA_strain,rows=c(1,3:7),
    mlab=mlabfun("RE Model for All Studies", res1), xlab = "Standardized Mean Difference",
    header="Author and Year - Time to Shedding")

### set font expansion factor (as in forest() above) and use a bold font
op <- par(cex=0.9, font=2)

### add additional column headings to the plot
text(c(-6.5,-5.0,-3.5), 8, c("N", "Age", " MDI"))
text(c(-5.0), 9, c("Challenge"))
text(c(0),9,c("Favors no sow Vx <--  --> Favors sow Vx     "))

### fit random-effects model in the three subgroups
res1.m <- rma(yi, vi, subset=(MDA_strain=="hom"), data=dat1shed)

### add summary polygons for the three subgroups
addpoly(res1.m, row= 2.0, cex=0.8, mlab=mlabfun("RE Model for Subgroup", res1.m))

### fit meta-regression model to test for subgroup differences
res1 <- rma(yi, vi, mods = ~ MDA_strain, data=dat1shed)

#################################################################
### Repeat for time to stop shedding
#################################################################
```

Step06 - Virus detection in nasal swabs
Meta-analysis using R metaphor package (metabin function)
Effect size = RR (Risk Ratio)
Input data = extracted as treatment group 2x2 table (binary data)
Details on meta-analytical method: - Mantel-Haenszel method, Restricted maximum-likelihood estimator for tau^2, Q-profile method for confidence interval of tau^2 and tau, Continuity correction of 0.5 in studies with zero cell frequencies.

```
setwd("C:/Users/sheila/Documents/PhD/Chapters/Chapter_3_data")

## load required package
library(metafor)
library(meta)
library(graphics)
library(ggplot2)

vpdata<-read.csv("vpos_MA_data.csv", header=T)

head(vpdata)
dim(vpdata)
str(vpdata)
```

```
vpdata.ma<-metabin(event.e, n.e, event.c, n.c,data=vpdata, sm="RR", method="MH", comb.random=F,
studlab=paste(Author, Year, sep=",  "))
summary(vpdata.ma)
forest(vpdata.ma)

#####repeat with sub-group analysis by MDA_Strain

vpdata<-read.csv("vpos_MA_data.csv", header=T)

head(vpdata)
dim(vpdata)
str(vpdata)

vpdata.mar<-metabin(event.e, n.e, event.c, n.c,data=vpdata, sm="RR", method="Inv",subgroup =
MDI_strain, studlab=paste(Author, Year, sep=","))

summary(vpdata.mar)
forest(vpdata.mar,leftcols = c("studlab", "Chal_age","PgVx_strain", "event.e","n.e", "event.c",
"n.c"),leftlabs = c("Study", "Chal_age", "PgVx", "Pos", "Tot", "Pos", "Tot"),addrows=2, label.right="Favors
no Sow Vx", label.left="Favors Sow Vx")
```

Step07 – Average daily gain (ADG)
Meta-analysis using R metaphor package (escalc & rma functions)
Effect Size = MD (Mean Difference)
Input Data = group size, mean, and standard deviation (as continuous data)

- extracted mean, sd, and n for each group as tabled in DeBlanc – all studies from Deblanc.   No other ADG results reported

```
setwd("C:/Users/sheila/Documents/PhD/Chapters/Chapter_3_data")


## load required package
library(metafor)
library(meta)
library(graphics)
library(ggplot2)

#####  analysis of mean differences in average daily gain during the 48 hrs post challenge using
### treatment group size, mean and sd provided in Ref id 3056 Table 2 pg 146.

setwd("C:/Users/sheila/Documents/PhD/Chapters/Chapter_3_data")

adg1md<-read.csv("adg_md_exp1.csv", header=T)

head(adg1md)
dim(adg1md)
str(adg1md)

#### inputs from data extraction= sd and mean from raw data and then to apply shim et al code for using
escalc
```

```
#### to derive md and variance (yi and vi) for meta-analysis input and forest plot generation.

datadg1 <- escalc(measure="MD", m1i=m1i, sd1i=sd1i, n1i=n1i,
            m2i=m2i, sd2i=sd2i, n2i=n2i, data=adg1md)
datadg1

adg1 <- rma(yi,vi,data=datadg1, slab=paste(Author, Year, sep=",  "))
adg1
adg1$I2
confint(adg1)
summary (adg1)
influence(adg1)

### a helper function to add Q-test, I^2, and tau^2 estimate info
mlabfun <- function(text, adg1) {
  list(bquote(paste(.(text),
            " (Q = ", .(formatC(adg1$QE, digits=2, format="f")),
            ", df = ", .(adg1$k - adg1$p),
            ", p ", .(metafor:::.pval(adg1$QEp, digits=2, showeq=TRUE, sep=" ")), "; ",
            I^2, " = ", .(formatC(adg1$I2, digits=1, format="f")), "%, ",
            tau^2, " = ", .(formatC(adg1$tau2, digits=2, format="f")), ")")))}


### set up the forests plot (with extra rows of information about)
forest(adg1,showweights=TRUE,ilab=cbind(datadg1$tot_n, datadg1$Chal_age, datadg1$Exposure),
    ilab.xpos=c(-6.5,-5.0,-3.5), xlim=c(-11, 5.5),cex=1, ylim=c(-1.5, 9),alim = c(-1,1), order =
datadg1$Exposure,rows=c(1:4),
    mlab=mlabfun("RE Model for All Studies", adg1), xlab = "Mean Difference",
    header="Author and Year - ADG (kg) 48 hr post-challenge (first exposure)")

### set font expansion factor (as in forest() above) and use a bold font
op <- par(cex=0.9, font=2)

### add additional column headings to the plot
text(c(-6.5,-5.0,-3.5), 5, c("N", "Age", " Exposure"))
text(c(-5.0), 6, c("Challenge"))
text(c(0),6,c("MDI increases effect <--    --> MDI decreases effect     "))


##############################################################
###############################################################
### Repeat for groups challenged a second time (x2)

#####  analysis of mean differences in average daily gain during the 48 hrs post 2nd challenge using
### treatment group size, mean and sd provided in Ref id 3056 Table 2 pg 146.
###  Deblanc exposed two groups twice - can't directly combine with first exposure effect sizes as 2nd
exposure ###animals are the same 1st exposure groups
### and are therefore not independent - they are correlated [ r ]

###Need to factor in r into estimates of mean and sd as correlated with first exposure findings if
combining in a ###single MA.

adg2md<-read.csv("adg_md_exp2.csv", header=T)

head(adg2md)
dim(adg2md)
```

```
str(adg2md)
datadg2 <- escalc(measure="MD", m1i=m1i, sd1i=sd1i, n1i=n1i,
              m2i=m2i, sd2i=sd2i, n2i=n2i, data=adg2md)
datadg2

adg2 <- rma(yi,vi,data=datadg2, slab=paste(Author, Year, sep=",  "))
adg2
adg2$I2
confint(adg2)
summary (adg2)
influence(adg2)

### a helper function to add Q-test, I^2, and tau^2 estimate info
mlabfun <- function(text, adg1) {
  list(bquote(paste(.(text),
                " (Q = ", .(formatC(adg1$QE, digits=2, format="f")),
                ", df = ", .(adg2$k - adg2$p),
                ", p ", .(metafor:::.pval(adg2$QEp, digits=2, showeq=TRUE, sep=" ")), "; ",
                I^2, " = ", .(formatC(adg2$I2, digits=1, format="f")), "%, ",
                tau^2, " = ", .(formatC(adg2$tau2, digits=2, format="f")), ")")))}


### set up the forests plot (with extra rows of information about exposure and challenge age)
forest(adg2,showweights=TRUE,ilab=cbind(datadg2$tot_n, datadg2$Chal_age, datadg2$Exposure),
      ilab.xpos=c(-6.5,-5.0,-3.5), xlim=c(-11, 5.5),cex=1.2, ylim=c(-1, 6),alim = c(-1,1), order =
datadg2$Exposure,rows=c(1:2),
      mlab=mlabfun("RE Model for All Studies", adg1), xlab = "Mean Difference",
      header="Author and Year - ADG (kg) 48 hr post-challenge (2nd exposure)")

### set font expansion factor (as in forest() above) and use a bold font
op <- par(cex=0.9, font=2)
### add additional column headings to the plot
text(c(-6.5,-5.0,-3.5), 3, c("N", "Age", " Exposure"))
text(c(-5.0), 3.5, c("Challenge"))
text(c(0),3.5,c("MDI increases effect <--    --> MDI decreases effect     "))
```

Step08_V2 – Hemagglutinin Inhibition titres (HAI)
Forest plot using R metaphor package (escalc & forest functions)
Effect Size = MD (Mean Difference in reciprocal log2 HAI GMT between baseline MDA+ arm versus MDA- arm)
Input Data = group size, mean, and standard deviation (as continuous data)

```
setwd("C:/Users/sheila/Documents/PhD/Chapters/Chapter_3_data")

## load required package
library(metafor)
library(meta)
library(graphics)
library(ggplot2)

##### analysis of mean differences in hai baseline titres at challenge in mda pos versus mda neg
###using data extracted manually (mean titres and error sd error bars) using webplot digitizer into excel
### titres = reciprocal log 2 GMT
```

```
hai1md<-read.csv("hai_md_pos_neg_bl.csv", header=T)

head(hai1md)
dim(hai1md)
str(hai1md)

#### inputs from data extraction= sd and mean from raw data and then to apply shim et al code for using
escalc
#### to derive md and variance (yi and vi) for meta-analysis input and forest plot generation.

dathai1 <- escalc(measure="MD", m1i=m1i, sd1i=sd1i, n1i=n1i,
              m2i=m2i, sd2i=sd2i, n2i=n2i, data=hai1md)
dathai1
##########################################################################
##########################################################################

### set up the forests plot (with extra rows of information about)
forest(hai1,showweights=FALSE,ilab=cbind(dathai1$tot_n, dathai1$MDA_strain, dathai1$Chal_age,
dathai1$Exposure), addfit=FALSE,
     ilab.xpos=c(-14.5, -13.5,-11.5,-9.5), xlim=c(-21, 11.5),cex=1, ylim=c(1, 10),alim = c(-7,6), order =
dathai1$Exposure,rows=c(1:5),
      xlab = "Mean Difference",
     header="Author and Year - HAI (GMT) MD in MDI+ vs MDI- baseline titre at challenge")

### set font expansion factor (as in forest() above) and use a bold font
op <- par(cex=0.9, font=2)

### add additional column headings to the plot
text(c(-14.5, -13.5,-11.5,-9.5), 6, c("N", "MDI", "Age"," Exposure"))
text(c(-11.5), 7, c("Challenge"))
```
