# Supplementary material for: Does Vaccine-Induced Maternally-Derived Immunity Protect Swine Offspring against Influenza a Viruses? A Systematic Review and Meta-Analysis of Challenge Trials from 1990 to May 2021
Source: Animals (Basel). 2023 Oct 3;13(19):3085. doi: 10.3390/ani13193085 (PMC10571953; doi:10.3390/ani13193085)
Supplement: Supplementary file 1 [file animals-13-03085-s001.zip › Supplemental files/S5 Fig.pdf]

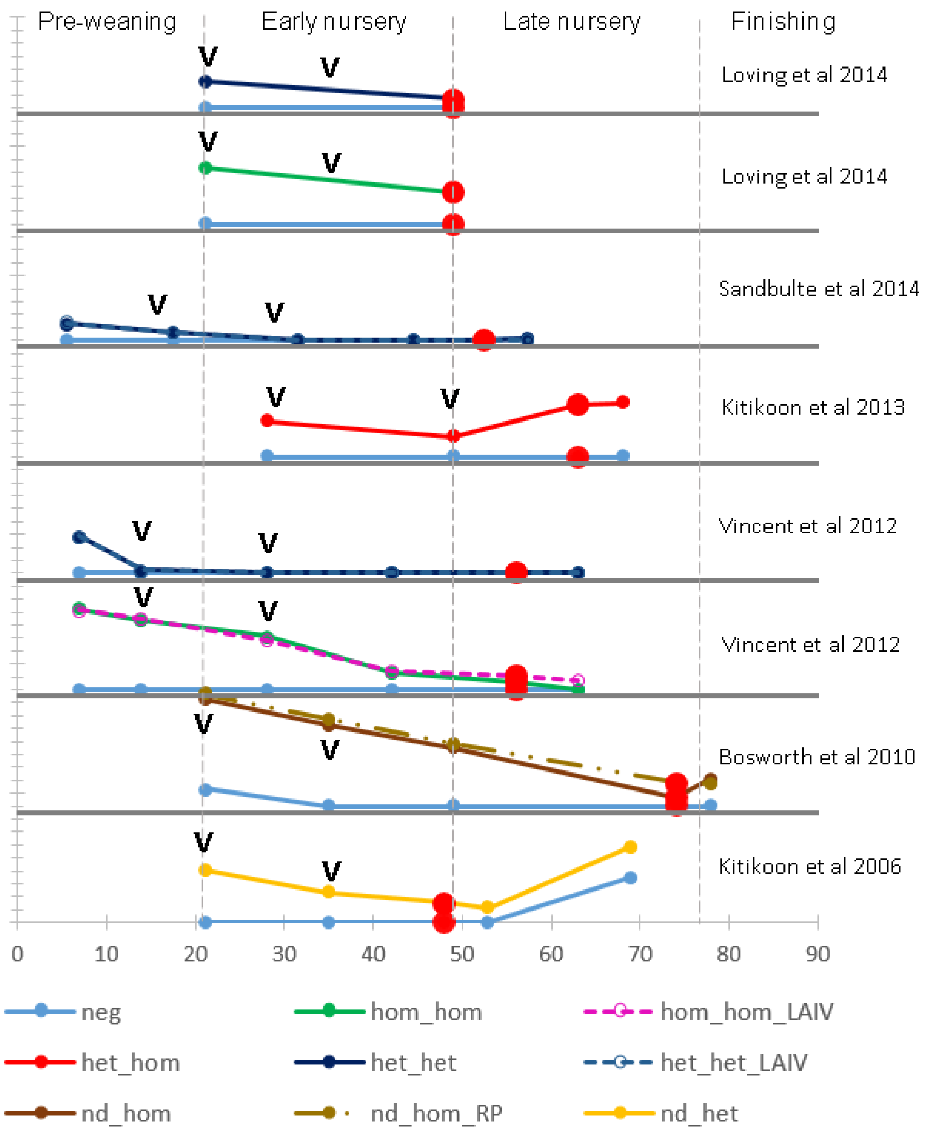

Figure S5. Bar graph, stacked by challenge trials, comparing group mean hemagglutination inhibition (HI) titres at each sample collection time-point (days of age) for MDI negative versus MDI positive and concurrently IAV-S vaccinated piglets for each of 6 IAV-S vaccine-induced MDI challenge trial.

Ordered by publication date; MDI positive = piglets with vaccine-induce maternally-derived immunity; MDI negative = control piglets from unvaccinated and IAV-S negative sows; MDI homology = antigenic match of the maternal vaccines with the challenge virus strain (heterologous or homologous); HAI assay antiserum is against challenge virus; x axis =piglet days of age, y-axis (unlabelled) = Log2 reciprocal geometric mean titre (GMT) with stacked studies each on the same scale of 0 to 9 (equates to HI titres <1:10 to 1:2560 where 3 = protective titre of 1:40); grey vertical dashed lines equal 3, 7 and 11 weeks of age; sow vaccines were HA sub-type homologous with challenge strain but varied by strain homology as indicated except Kitikoon (2006) where the sow vaccine was not defined; vaccines were whole killed virus (WIV) vaccines unless otherwise indicated where RP = replicon particle and LAIV = live attenuated influenza virus; **V** indicates timing of piglet IAV-S vaccination; red circles indicate the timing of piglet challenge; hom, het, and nd = vaccine strain homologous, heterologous, or not defined, respectively, in reference to the challenge virus strain; neg = MDI negative; legend entrees listed as homology of sow vaccine_homlogy of piglet vaccine_ piglet vaccine platform type.
