# Supplementary material for: Does Vaccine-Induced Maternally-Derived Immunity Protect Swine Offspring against Influenza a Viruses? A Systematic Review and Meta-Analysis of Challenge Trials from 1990 to May 2021
Source: Animals (Basel). 2023 Oct 3;13(19):3085. doi: 10.3390/ani13193085 (PMC10571953; doi:10.3390/ani13193085)
Supplement: Supplementary file 1 [file animals-13-03085-s001.zip › Supplemental files/S5 Text.pdf]

S5 Text: Serum Hemagglutination Inhibition (HI) Titres (qualitative synthesis) - Indirect measure of protection: Additional explanation.

In study arms with MDI positive piglets [85–87,92] post-vaccination serologic responses were absent in all groups with the exception of Kitikoon (2013)[87] (S5 Fig). Baseline mean HI titres in MDI positive groups at the time of piglet vaccination exceeded a titre of 1:40 except, notably in the Kitikoon 2013 MDI positive group where the baseline titre was < 1:40 at the time of booster (***S5*** Fig).

Two studies involved a repeated challenge of piglets (Deblanc) [89] (S4 Fig); none of the piglets was vaccinated against IAV-S and both challenges were with the same virus that was also subtype and strain homologous to the viral antigens in the maternal vaccine. None of the MDI negative nor MDI positive piglets became infected upon secondary challenge. Using individual pig data available from the above studies and a third by the same authors using the same challenge virus and vaccine (i.e. antigenically subtype and strain homologous) [89]), differences in mean HI titres at the time of challenge (i.e. baseline titres) between MDI positive versus MDI negative groups were calculated and are shown as a forest plot in S4 Fig. On first exposure, MDI positive piglets had significantly greater baseline mean HI titres than MDI negative piglets at weaning. Difference in baseline titres diminished with increasing age at first challenge, and was non-significant for piglets challenged at 11 weeks of age. A similar diminishing trend was apparent between MDI positive and negative groups upon secondary challenge, however, the direction was reversed where instead mean baseline HI titres were greater for MDI negative versus MDI positive piglets (S6 Fig).
