# Supplementary material for: Does Vaccine-Induced Maternally-Derived Immunity Protect Swine Offspring against Influenza a Viruses? A Systematic Review and Meta-Analysis of Challenge Trials from 1990 to May 2021
Source: Animals (Basel). 2023 Oct 3;13(19):3085. doi: 10.3390/ani13193085 (PMC10571953; doi:10.3390/ani13193085)
Supplement: Supplementary file 1 [file animals-13-03085-s001.zip › Supplemental files/S6 Fig.pdf]

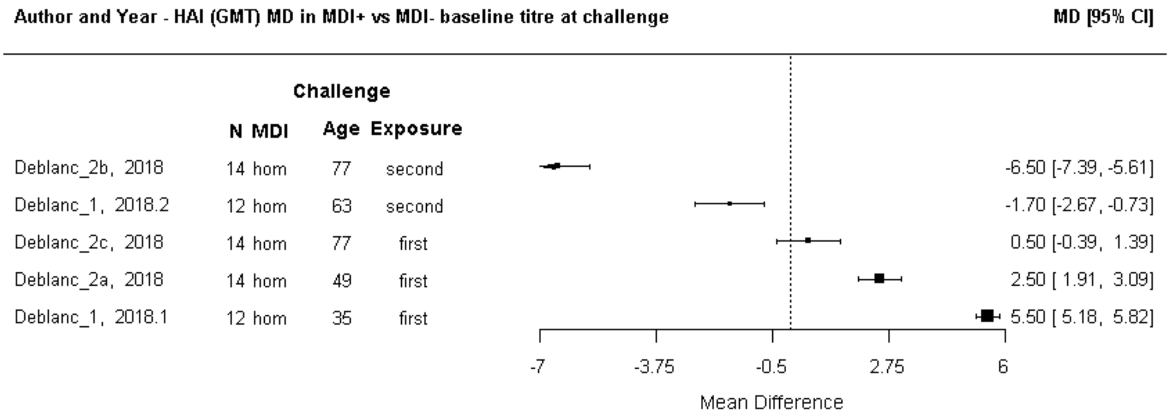

Figure S6. Forest plot of effects of IAV-S vaccine-induced MDI on the difference in mean HI titres at challenge between MDI positive vs MDI negative piglets at time of first and second IAV-S challenge.

Effect is the HI baseline titre (log2 reciprocal geometric mean titre) at time of challenge for each of MDI positive and MDI negative piglets; MDI = antigenic match of the maternal vaccine composition to the challenge virus (heterologous (het) or homologous (hom)); MDI = IAV-S vaccine induced maternally-derived immunity; MDI positive = offspring of IAV-S vaccinated dams; MDI negative = control groups of offspring from non-vaccinated and IAV-S negative control dams; baseline measure taken at 4 dpc in the first exposure trials and at 1 dpc in the second exposure trials; effect size is difference in mean titres between the MDI positive versus MDI negative control groups at the time of challenge; the dotted vertical line indicates a mean difference of 0 (no difference in baseline titres between groups at the time of challenge) and points to the right of the line indicates higher baseline titres in MDI positive pigs vs MD negative pigs.
