# Supplementary material for: Does Vaccine-Induced Maternally-Derived Immunity Protect Swine Offspring against Influenza a Viruses? A Systematic Review and Meta-Analysis of Challenge Trials from 1990 to May 2021
Source: Animals (Basel). 2023 Oct 3;13(19):3085. doi: 10.3390/ani13193085 (PMC10571953; doi:10.3390/ani13193085)
Supplement: Supplementary file 1 [file animals-13-03085-s001.zip › Supplemental files/S6 Text.pdf]

S6 Text. VEARD, IAV-S vaccine-induced MDI, and IAV-S vaccines in swine challenge trials: a narrative summary (Direct measures of infection – non-specific endpoint).

Vaccine associated enhanced disease is observed across species in association with vaccines against different pathogens and is an important obstacle in human and veterinary vaccine development [30,116]. In swine it manifests as a severe lung pathology and is called vaccine associated enhanced respiratory disease (VAERD). Although mechanisms of VAERD are not fully understood, aspects of cross-reacting but non-neutralizing, and possibly fusion-enhancing antibodies are believed important [13,134]. Inducing conditions include vaccination with homosubtypic but strain heterologous whole inactivated vaccines (WIV) [13,35,115–117], with a strain heterologous HA1 sub-unit vaccine (suggesting HA mismatch alone as a cause) [36], and with a candidate M2e fusion protein DNA vaccine [135]. Across the body of evidence all researchers expressed concerns with use of pre-farrowing WIV vaccines due to piglet impaired immune responses and/or potential for increased risk of VEARD. Almost half of all eligible studies (n=7), and an additional study which met all eligible criteria except measure of review outcomes, described assessment of VEARD as a study objective, of which, all but one included also vaccination of piglets against IAV-S. Study details are tabled below.

Summary details of studies investigating VAERD as a stated objective:

Author (1st)	Affiliation[Ŧ]	Piglet vaccine[Ŧ]	Study length (dpc)
†Kitikoon 2006 [69]	ISU	yes	21
Vincent 2012 [86]	ARS/USDA	yes	5
Kitikoon 2013 [87]	ARS/USDA	yes	5
Sandbulte 2014 [90]	ARS/USDA	yes	8
Loving 2014 [92]	ARS/USDA	yes	5
Rajao 2016 [88] (2 studies)	ARS/USDA	no	17
◊Pyo 2015 [83]	VIDO	yes	5

Ŧ Organizational Affiliation of corresponding author ; Ŧ Research primary objective was investigation of efficacy of IAV-S vaccination of offspring; dpc = days post-challenge; †Investigation of vaccine associated enhanced respiratory disease (VEARD) not a stated objective but this is an important early pig study describing findings consistent with VEARD; ◊ Pyo (2015) study met all inclusion criteria but did not report outcomes of interest for this review (i.e. not eligible for inclusion but included for completeness of IAV-S vaccine induced MDI challenge studies); ISU= Iowa State University; ARS/USDA = Agricultural Research Service of the United States Department of Agriculture; VIDO = Vaccine and Infectious Disease Organization; cells shaded in grey or orange indicate studies funded by governmental organizations in the U.S.A or Canada, respectively

In 2006, Kitikoon [69] observed enhanced disease in piglets from IAV-S vaccinated dams -and cautioned against use of maternal IAV-S vaccines. Subsequently researchers at USDA's Virus and Prior Research Unit identified increased risk of adverse vaccine outcomes in swine following introduction of new influenza sub-types and strains into the North American swine population. They established a pig model to induce vaccine enhanced acute respiratory disease (VEARD) where pigs vaccinated with antigenically mismatched whole inactivated vaccines (WIV) produced subtype homologous, but strain heterologous antibodies to the challenging virus [35]. Vincent [86], Kitikoon[87], and then Sandbulte [90] subsequently demonstrated VEARD occurred in MDI positive pigs, and in MDI positive and concurrently WIV vaccinated pigs (both maternal and piglet vaccines heterologous, see Table 2, combination No. 13). Loving[92] demonstrated cross-fostering was not a condition of VEARD, and finally, Rajao [88] observed VEARD was reproduced in unvaccinated piglets with MDI from sows vaccinated with a heterologous WIV, but was not reproduced when the MDI was from sows naturally or experimentally infected with heterologous wild-type viruses.

Interestingly, observation of VEARD by Sandbulte [90] differed from those by Vincent [86] where no increased propensity was observed in MDA positive pigs despite use of the same antigenic heterologous combination of maternal and piglet vaccines with challenge viruses (see S3 Table and S4 Table). Differences in HI titres at the time of challenge, and differences in challenge dose and method of delivery were considered by the author as possible explanations.

Additionally, in this study less than half of the pigs in the MDA control group (3/8) became infected which differed from other challenge studies. Cador [89] reported a dose response relation between the level of baseline vaccine-induced MDI following uptake of colostrum with the piglet's response to challenge [93].

Determining the prevalence of VEARD in commercial swine populations will be challenging. In swine populations consideration of multiple factors is required including concurrent infections with pathogens involved in porcine respiratory disease complex (PRDC) [136], and factors affecting influenza virus kinetics in farms such as individual animal immunity, the number and homology of virus strains in circulation, and the frequency, timing, and infection status of animal introductions [37,137].
