# Supplementary material for: Does Vaccine-Induced Maternally-Derived Immunity Protect Swine Offspring against Influenza a Viruses? A Systematic Review and Meta-Analysis of Challenge Trials from 1990 to May 2021
Source: Animals (Basel). 2023 Oct 3;13(19):3085. doi: 10.3390/ani13193085 (PMC10571953; doi:10.3390/ani13193085)
Supplement: Supplementary file 1 [file animals-13-03085-s001.zip › Supplemental files/S7 Fig.pdf]

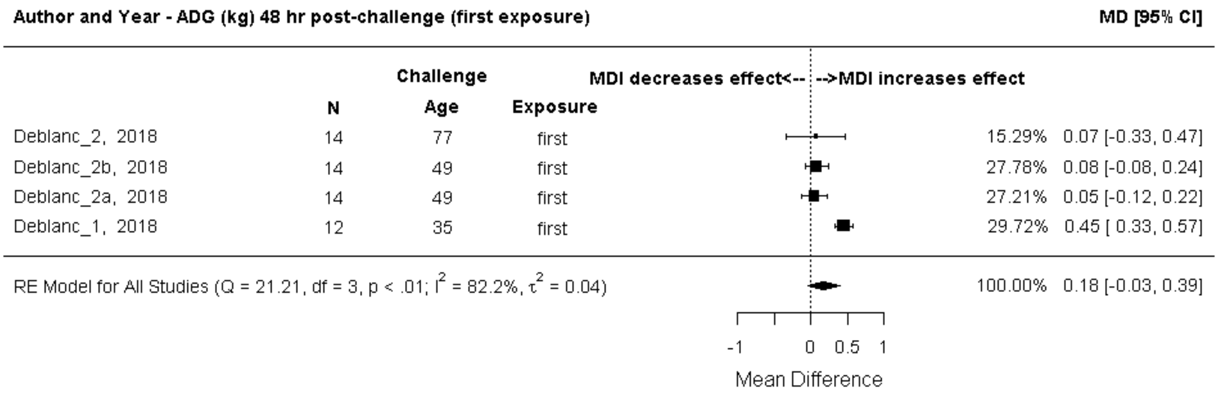

Figure S7. Random effects meta-analysis forest plot of effects of vaccine-derived MDI on the mean difference ADG (kg) in IAV-S challenged piglets following 1st challenge.

ADG=average daily gain; effect is the mean weight gain (kg) during the 48 hours post-challenge (dpc); MDI = vaccine induced maternally derived immunity where the antigenic match of the maternal vaccine composition to the challenge virus was homologous for all comparisons); effect size is difference in the mean effect in MDI positive treatment groups versus MDI negative control groups; the dotted line indicates a mean difference of 0 (no difference between MDI positive and MDI negative groups); points to the right indicates MDI increases effect. I^2 95% uncertainty interval (lower bound, upper bound) = (44.43, 98.47).
