# Supplementary material for: Does Vaccine-Induced Maternally-Derived Immunity Protect Swine Offspring against Influenza a Viruses? A Systematic Review and Meta-Analysis of Challenge Trials from 1990 to May 2021
Source: Animals (Basel). 2023 Oct 3;13(19):3085. doi: 10.3390/ani13193085 (PMC10571953; doi:10.3390/ani13193085)
Supplement: Supplementary file 1 [file animals-13-03085-s001.zip › Supplemental files/S7 Table.pdf]

S7 Table. GRADE Partial† evidence profile: IAV-S vaccination of sows for the protection of offspring against IAV-S challenge

Outcome	Limitations (Risk of Bias)‡	Inconsistency † †	Indirectness	Imprecision	Publication Bias	No. MDI –'ve offspring (control)	No. MDI +'ve offspring (treatment)	No. of studies
Virus detection (incidence)	No serious limitations	No serious inconsistency	↓ by 1 level serious indirectness[b]	N/D	Not determined but unlikely*	98	142	5
Virus titres	No serious limitations	NA	↓ by 1 level serious indirectness[b]	N/D	None detected	96	171	8
Virus shedding								
i)Detection of shedding	No serious limitations	No serious inconsistency	↓ by 1 level serious indirectness[b]	N/D	Not determined but unlikely *	90	110	4
ii)Days to begin shedding:	No serious limitations	NA	↓ by 1 level serious indirectness[b]	N/D	Not determined but unlikely *	90	110	4
iii)Ceasing to shed	No serious limitations	No serious inconsistency	↓ by 1 level serious indirectness[b]	N/D	Not determine but unlikely *	90	91	4
iv)Days of shedding	No serious limitations	NA	↓ by 1 level serious indirectness[b]	N/D	Not determined but unlikely *	90	91	4
HI titre	No serious limitations	NA	↓ by 1 level serious indirectness[b]	N/D	Not determined but unlikely *	20	20	2
ADG	No serious limitations	NA	↓ by 1 level serious indirectness[b]	N/D	Not determined but unlikely *	27	27	2
Coughing	↓ by 1 level [a] (Serious limitation)	↓ by 1 level[c] serious inconsistency	↓ by 1 level serious indirectness[b]	N/D	Not determined but unlikely *	24	24	1

GRADE=Grading of Recommendations Assessment, Development, and Evaluation [77]; †Imprecision not assessed and overall GRADE score not assigned; †† inconsistency assessment applied to relative measures only, not applicable to absolute measures of effect; ‡See Risk of Bias assessment S5 Table; * Too few studies to construct a funnel plot, majority of research was government funded; N/A = not applicable; N/D = not done

[a] criteria for coughing measure not defined; [b] challenge trials poorly extrapolated to field conditions (i.e. unnatural exposure conditions, short study time periods, unreported or no comorbidities, small population sizes); [c] based on a single study effect size calculated from extracted raw data, RR not significant for association of cough to MDI status.
