# Supplementary material for: Does Vaccine-Induced Maternally-Derived Immunity Protect Swine Offspring against Influenza a Viruses? A Systematic Review and Meta-Analysis of Challenge Trials from 1990 to May 2021
Source: Animals (Basel). 2023 Oct 3;13(19):3085. doi: 10.3390/ani13193085 (PMC10571953; doi:10.3390/ani13193085)
Supplement: Supplementary file 1 [file animals-13-03085-s001.zip › Supplemental files/S7 Text.pdf]

S7 Text. Outcome 5. Mean difference (MD) in average daily gain: Additional explanation **(Direct measure of infection – non-specific endpoint).**

Pigs in a single treatment-control comparison group in each of the studies were challenged a second time 4 weeks later and the mean difference in ADG was significantly less in pigs with MDI versus without (MD=-0.40 kg/day, 95%CI:-0.61,-0.19) when the second challenge was at 77 days of age but mean difference was not significant in comparison groups challenged at 63 days of age (MD=-0.18 kg/day, 95%CI:-0.38-0.02).
