# Supplementary material for: Does Vaccine-Induced Maternally-Derived Immunity Protect Swine Offspring against Influenza a Viruses? A Systematic Review and Meta-Analysis of Challenge Trials from 1990 to May 2021
Source: Animals (Basel). 2023 Oct 3;13(19):3085. doi: 10.3390/ani13193085 (PMC10571953; doi:10.3390/ani13193085)
Supplement: Supplementary file 1 [file animals-13-03085-s001.zip › Supplemental files/S8 Text.pdf]

S8 Text. Outcome 6. Coughing (insufficient data for meta-analysis):

Additional explanation (Direct measure of infection – non-specific endpoint).

In (Kitikoon,2006) 69], MDI positive piglets (including treatment groups with both vaccinated and unvaccinated piglets) were 2.3 times more likely to cough in the 7 days post-challenge than piglets without MDI (RR=2.3, 95%CI: 2.0-2.7). Of the MDI positive piglets, those that were also vaccinated were 2.9 times as likely to cough as compared to MDI negative piglets (RR=2.9, CI: 2.3-3.8). The risk of MDI positive and unvaccinated piglets was twice that of MDI negative piglets (RR = 2, CI: 1.7-2.4). In this study the sow's prior IAV- S exposure was undefined and the piglet WIV vaccine was strain heterologous to challenge virus (see Table 2, Combination No. 7). Sandbulte [90] reported coughing narratively as part of a composite score of lethargy, labored breathing and coughing but scoring data for non-aggregated parameters were not provided. Respiratory distress was greatest in heterologous MDI positive piglets that were concurrently vaccinated with strain heterologous WIVs (see Table 2. Combination 13). In Sandbulte [90], dam vaccines were also strain heterologous with respect to the challenge virus.
